# Use of the “Future Life Map” exercise to improve awareness of career options and opportunities in underrepresented minority undergraduate students pursuing STEM careers

**DOI:** 10.1371/journal.pone.0263848

**Published:** 2022-02-10

**Authors:** Anne Whitehead, Nathan J. Alves

**Affiliations:** Department of Emergency Medicine, Indiana University School of Medicine, Indianapolis, IN, United States of America; University of Texas Southwestern Medical Center at Dallas, UNITED STATES

## Abstract

**Objectives:**

There has long existed significant underrepresentation of minority students in STEM training and careers. Ongoing efforts to improve opportunities and participation for underrepresented minority students have focused on multiple areas, from increased funding to early exposure to research in STEM. We developed the novel Future Life Map career planning exercise with the goal of contributing to this multi-faceted approach. The exercise emphasizes on the consideration of multiple potential career destinations and routes to those destination. The exercise was designed with the goal of improving participant awareness of options and career planning self-efficacy to improve success and retention of underrepresented minority student participation and retention in STEM.

**Methods:**

We implemented the Future Life Map exercise with 2 separate groups of under-represented minority undergraduate students pursuing careers in STEM. Participants then completed an anonymous survey to evaluate the exercise and describe the value they derived from completing the Future Life Map.

**Results:**

The exercise presentation and its supporting documents were highly rated by participants with >81% of respondents rating it as “very informative” (4 or 5 on a 5-point Likert Scale). Participants reported that they were very likely to recommend the exercise to others (25 of 27 participants) and were likely to repeat the activity for their own future decision making (22 participants). Themes that emerged from participant reporting of the value of the exercise were: increased awareness of career and training options, improved understanding of the research required to make informed career/life decisions, and new awareness of specific information about career options under consideration.

**Conclusion:**

The Future Life Map exercise was successful in improving participant awareness of career options, career planning ability, and helped participants to feel more empowered. This is likely of particular benefit for improving participation and retention of under-represented minority students pursuing careers in STEM.

## Introduction

Despite considerable efforts to improve diversity in science, technology, engineering, and math (STEM) degree programs and careers, there has long been, and remains significant underrepresentation of minorities, specifically black and latinx, in the field of STEM [1, 2]. Many programs have been created to correct this under-representation, largely through the recruitment of under-represented minority (URM) students to STEM fields. These recruitment efforts have focused on early exposure to STEM research and other experiences, funding to address resource gaps that disproportionately affect URMs, and mentorship programs [3–5]. Study of these programs has highlighted elements that are associated with improved success in recruitment and retention of URMs in STEM. These elements include: focus on improving self-efficacy, matching fields of study with URM student and scientist values, and strong mentorship. It was with these important elements in mind that we designed and implemented the “Future Life Map” activity to be used as a tool for improving URM recruitment to and retention in STEM fields.

The process of “career mapping” or “career cartography” has long been a tool used in aiding individuals from all backgrounds in achieving career success. These exercises involve the creation of a visual depiction of long-term career goals, and the steps to achieve these goals. While a variety of models exist, they typically employ a stepwise approach, encouraging first an in-depth exploration of values and interests which then leads to the identification of a singular career goal [6–8]. After this goal is selected, then requirements and steps to achieve this goal are researched and diagrammed. This approach emphasizes one, or a small number of “destinations” or final goals, all of which the author of the map hopes to achieve. These models tend to limit broad exploration of opportunities and do not encourage the inclusion of alternate destinations or “back-up” plans. Often there is little emphasis placed on having the individual determine if the steps on the way to the end destination are in alignment with their values, interests, and/or available resources.

We designed and implemented the Future Life Map exercise as a broader, more open career mapping activity which encourages participants to consider multiple career options simultaneously. They are asked to envision pathways to a variety of career types and consider a variety of factors that might affect their choices of next steps including: cost, time spent in training, wellness, perceived value of work, and any other considerations they deem of importance or value to them. The objective of this activity is to empower participants with skills to make informed career decisions, consider a wider array of options than typical, and thereby improve career and life planning self-efficacy and resiliency.

We implemented this exercise with two cohorts of *Louis Stokes Alliances for Minority Participation* (LSAMP) undergraduate students (27 in total) pursuing careers in STEM, with the goal of contributing to improved recruitment and retention of URM students into STEM careers.

## Methods

### Participants

The Future Life Map Exercise was presented to two different cohorts of undergraduate students enrolled in *Louis Stokes Alliances for Minority Participation* (LSAMP) [9] programs in 2 different midwestern states, Indiana (IN-LSAMP) and Missouri (MO-LSAMP), and the participants included in this study totaled 27. This NSF sponsored program consists of ~56 active alliances nationally. The LSAMP program has an overall goal of assisting universities and colleges in diversifying the STEM workforce by increasing the number of STEM degrees awarded to those from populations historically underrepresented in STEM. The program lists these populations as African Americans, Hispanic Americans, American Indians, Alaska Natives, Native Hawaiians, and Native Pacific Islanders.

Participants begin LSAMP with a summer intensive program which consists of weekly didactic sessions, course work, and a mentored research experience. The Future Life Map exercise was presented to student participants in two back-to-back required weekly meetings. These sessions were one-hour in duration each and were all virtual due to the ongoing SARS-CoV-2 (COVID-19) pandemic.

### Exercise structure

**Fig 1:**
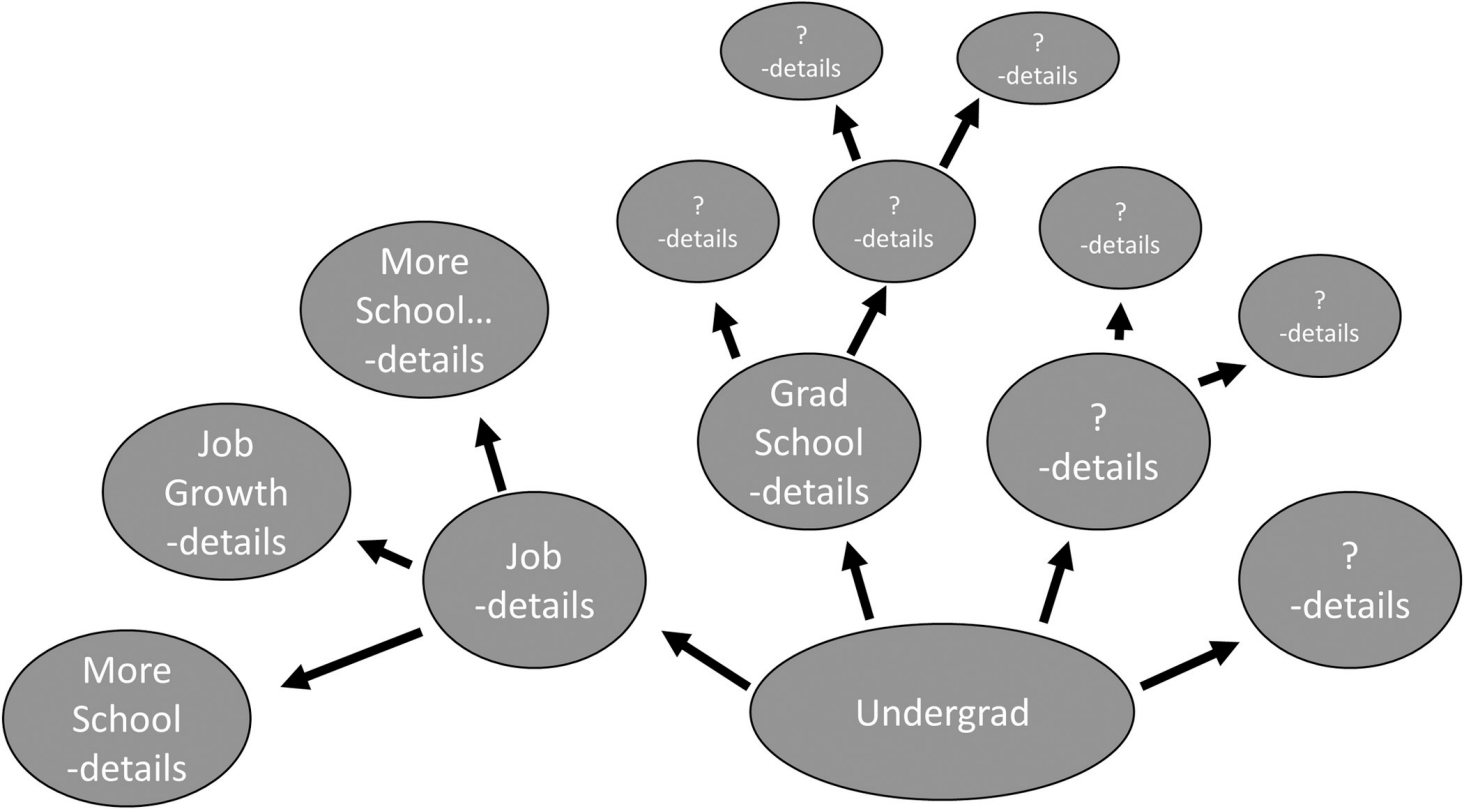
Future Life Map example template. This serves as a rough framework for participants, but they are encouraged to edit the template and be creative in organizing the different pathways as they see fit. More detailed instructions for carrying out the exercise included in S1 and S2 Appendices.

The session instructor contacted the participants via email 2 days before the initial zoom session. The instructor provided them with a written introduction to the session and instructions for completion of each participant’s own personal Future Life Map (S1 Appendix). This document included an empty future life map template to use for guidance (**Fig 1**). It encouraged participants to complete the exercise by hand on a large sheet of paper or poster board. The document instructed participants to start the map at their current career/training stage, then begin to draw multiple branches connecting to a variety of possible next steps, decision points, and opportunities. This structure of exploring, in detail, multiple discreet possible pathways, is a departure from the structure of prior career mapping exercises described in the literature. This allows the participant to avoid committing to a single career goal prior to fully understanding the available options, and the factors that might affect their decisions. It also allows the participant to use the insight and information gained during the exercise in the future, should they later need or desire a change in career path. This is a departure from prior exercises described that typically encourage career exploration be followed by a narrowing of options and the creation of a singular career pathway for the participant to attempt to follow. The document emphasized that even options in which they initially had less interest should be explored during the exercise to avoid premature closure of a career path not adequately understood by the participant. The instructions then asked that participants write benefits and drawbacks of each option on the map, as well as the demands, pre-requisites, associated deadlines, impact on wellness, impact on family, and any other factor they might consider important. The instructions asked participants to carry branches out as far as 10–20 years into the future.

Participants were instructed to familiarize themselves with the Future Life Map concept in preparation for the first session, but completion of their personal Future Life Maps was not expected until the second zoom session, which followed one week after the first session.

Each group participated in two one-hour sessions that took place in the summer of 2020. The two sessions were separated by 1 week for both groups. The first group had 14 participants, and the second, 13 participants, for a total of 27 participants included in the study. They were held during the COVID-19 pandemic prior to vaccine availability and as such were all held virtually utilizing the Zoom platform. Participants were requested, but not required, to have video active, and most participants opted to have video on. The instructor led the first session utilizing a PowerPoint slide deck (S2 Appendix). This instructor is involved in the LSAMP program at an institutional level but was not involved in the program directorship for participants in either group. The instructor discussed the goals and structure of the Future Life Map process to emphasize the need to cast a wide opportunity net and to do the research necessary to rule opportunities in or out. The instructor utilized chat and audio to engage learners and field any questions. The first portion of the session helped participants understand the principles and factors that should guide the creation of their own life maps. It encouraged participants to consider their own motivations and values. It emphasized the importance of researching multiple possible pathways to avoid missing opportunities. The second portion of the presentation walked the participants through the process of creating their own Future Life Maps. At the conclusion of the first session the instructor opened the discussion for questions and clarifications. This discussion included guidance from the instructor on strategies for researching career options including broad searching recommendations to help students (S1 Appendix: Tips for success in building your map). He then asked participants to complete their own future life maps independently in the time between the first and second sessions. He asked that they have them available during the second session.

Future Life Maps were checked for completion but were not collected. The instructor made the decision not to collect the maps as it was important that the exercise allow personal development in the absence of outside judgement. Having maps collected would likely have limited the openness and vulnerability with which the participants completed the exercise.

During the second one-hour session, no PowerPoint was used. The majority of the session consisted of open discussion. The instructor shared some examples from his own career planning and progression to start the session and help the audience feel more comfortable sharing. The instructor prepared and asked a series of open-ended questions to stimulate and guide the discussion (S3 Appendix). Participants were encouraged to share their answers to the open questions to the extent they were comfortable.

### Exercise evaluation

As the final activity of the second session, participants filled out a survey with questions pertaining to the strength of the presentation, the value of the exercise, and their comfort with planning for the future before and after the session (S4 Appendix). This was housed on a secure Google Forms survey. Results were collected anonymously, and none of the information gathered was shared with course directors or those responsible for evaluating the students.

### Data analysis and reliability

Both authors independently reviewed and categorized open ended participant responses into themes. They then compared the themes they identified and resolved any discrepancies in categorization via discussion. Statistical analysis was performed using Microsoft® Excel® for Microsoft 365. Likert scale questions were plotted based on percent respondents selecting each value. To investigate possible differences between the survey answers provided by male and female participants and for comparisons across linked questions a Chi-squared test was employed. A significance level of p<0.05 was used.

The Indiana University Institutional Review Board determined that this evaluation was exempt from review. Consent was not obtained as data were analyzed anonymously.

## Results

Twenty-seven LSAMP participants took part in the sessions, with 14 participants in one session and 13 in the other. The survey response rate was 100%. The mean(SD) age of participants was 20.8(3.1) years, with a range of 17 to 32 years old. Eight participants (34.8%) identified as male, 15 (55.6%) identified as female, and 4 (14.8%) did not identify a gender (**Table 1**). Only 4 participants (14.8%) reported having participated in a different career or life planning activity in the past. Most participants reported that the future life map exercise helped them to consider options they had not previously considered (88.9%) and/or to re-consider an option they had previously ruled out (74.1%). Most participants also reported that the exercise helped them learn more about their own motivations (85.2%).

**Table 1:** Responses from the Future Life Map survey separated by gender. Raw data provided in S5 Appendix.

Gender	Male	Female	No Response	Total
Subject (N)	8	15	4	27
Age [avg(stdev)]	22 (4.4)	20.1 (1.9)	--	20.8 (3.1)
Percent reporting previously performing a similar exercise	12.5	20	0	14.8
Percent reporting the exercise helped them to consider alternative options	87.5	86.7	100	88.9
Percent reporting the exercise helped them reconsider an option that was previously ruled out	62.5	73.3	100	74.1
Percent reporting learning new details about their motivation	75	86.7	100	85.2

**Fig 2:**
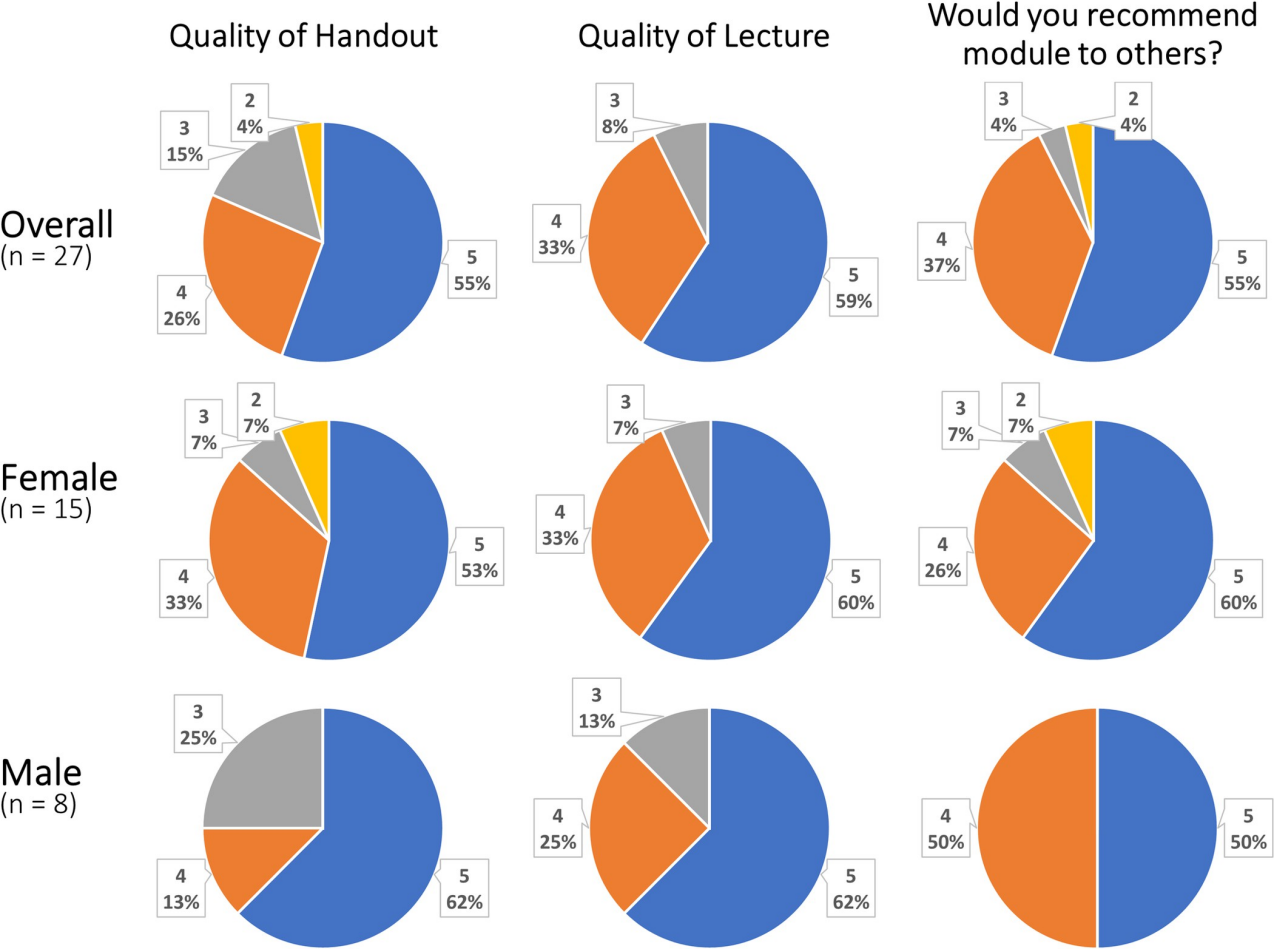
Participant rating of the components of the Future Life Map exercise. This shows the distribution of responses on 5-point Likert scales of the entire group, and separated by gender for those participants who identified a gender. Number indicates Likert score and percent corresponds to percent of respondents selecting that value.

Survey responses were split by gender and analyzed separately as well as in aggregate. Both the presentation and supporting document (S1 and S2 Appendices) were rated highly by participants on a 5-point Likert scale. They also reported being very likely to recommend the module to others with 92% of respondents scoring a 4 or 5 overall **(Fig 2)**.

There were largely no significant differences in the ratings provided by males vs females, with the exception of the question "how informed on future life decisions did you feel prior to performing the Life Map exercise?" For this question, males reported higher levels of being informed prior to the exercise than females (50% with a 4 or 5 rating for males, versus 13% with a 4 and no 5 ratings for females, p>>0.001). This difference did not persist for their ratings regarding how informed they felt after the exercise, with 88% and 93% rating 4 or 5 for males and females respectively. Additionally, there was a significant difference overall between the distributions of Likert scores of how informed participants felt before vs after the workshop (p = 0.0001). No other statistically significant differences were observed comparing male vs female responses to the Future Life Map exercise.

**Fig 3:**
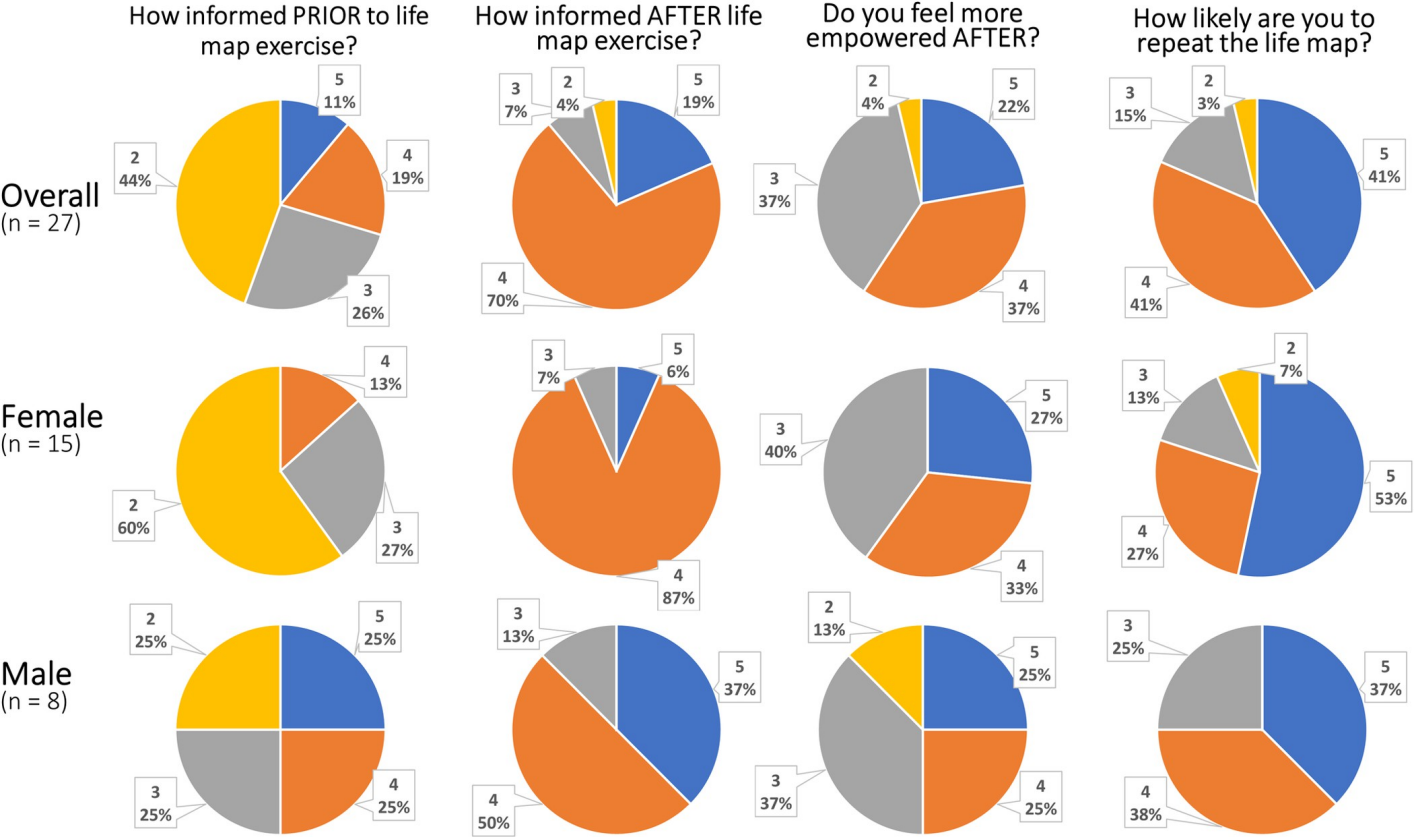
Participant rating of the outcomes of the Future Life Map exercise. This shows the distribution of responses on 5-point Likert scales of the entire group, and separated by gender for those participants who identified a gender. Number indicates Likert score and percent corresponds to percent of respondents selecting that value.

Overall, participants reported feeling more informed about future life decisions after the exercise than they were before. Nineteen of the 27 participants (70.4%) reported feeling more informed (**Fig 3**). Six (22.2%) reported feeling no change, and two (7.4%) reported feeling less informed after the exercise. The 2 participants who reported feeling less informed reported feeling more empowered about future life decisions (both 4 on a 5-point Likert scale). Participants also reported they were likely to repeat the activity for their own future decision making (additional analysis can be found in S5 Appendix).

In the survey, we asked participants to identify the most valuable part of the exercise. Several themes emerged. Twelve (44.4%) of the 27 participants reported becoming aware of a greater number of career and training options than they had previously considered and/or thought available to them. Eight (29.6%) of the participants reported it was valuable to realize how much more research might be required to make informed career selection decisions. Four (14.8%) reported finding value in learning specific information about career options they were considering. A sample of some, but not all, of the responses from participants describing the value of the exercise is included here (**Table 2**).

**Table 2:** Examples comments made by participants regarding the value of the Future Life Map exercise.

Theme	Examples Participant Comments
Increased awareness of career and training options	“I did not look into it as much as I should because honestly it scares me to think too far into the future, but this exercise made me reconsider.”
	“I was really stuck on one thing that I wanted to accomplish but looking at it now, it seems very possible I’ll be taking a different route than what I thought.”
	“Honestly I never realized how many options I really had in life. It was overwhelming but good that I put all of my options out there to see in person.”
Improved understanding of the research required to make informed career decisions	“I needed to do far more research into my future than I thought.”
	“I think looking at the actual job descriptions rather than labels was important. It "sounds" great to have a doctorate, but what does that actually mean? What opportunities are forfeited? I never thought about that.”
Learned specific information about career options under consideration	“I value the specific information he gave in the follow up session about the realities of a PhD vs Masters and medical school.”

Participants engaged well with the exercise and took ownership of the discussions. While the instructor prepared and asked a series of open-ended questions to stimulate and guide the discussion (S3 Appendix), many of the prepared questions were not needed during the sessions. It was common for the participants to ultimately guide the discussion to the challenges, discoveries, and questions that were of most interest and value to them. The topics discussed by participants varied between groups. It was also common for students to answer each other’s questions and provide peer-to-peer guidance.

## Discussion

### Educational value

The Future Life Map exercise was a highly rated, highly valued exercise among the participants. It was particularly successful in broadening participant’s understanding of the number of options they have in pursuing STEM training and careers. Many participant comments identify this increased awareness of options as particularly valuable in providing a “back-up plan” when barriers to progress are confronted, either as alternate routes to a particular goal, or as alternate destinations within STEM. One participant noted that the most valuable part of the exercise was “Knowing that there are different options that will get you to where you want to get.” This ability to easily identify an alternate route in the face of barriers, anticipated or unanticipated, likely improves resiliency for participants. The pipelines for URM students into STEM careers can contain many possible “exit points” often as a result of challenges and barriers within a set pathway to success [10]. Systemic and structural racism often make barriers more prevalent and more severe for URM students pursuing STEM careers. Combating systemic racism should be of high priority; however, providing tools for resilience like the Future Life Map exercise is still of importance in improving URM representation in STEM careers.

Similarly, participant responses suggest the exercise also contributes to improved self-efficacy in career planning. Increased awareness of options seemed to empower participants. While the exercise clearly improved participants’ understanding of options available, they also reported that they had an improved understanding of their own motivations, as well as an awareness of the research and attention to detail needed to make informed decisions. In effect, the exercise seemed to improve participant competency and self-efficacy in career planning. This is important in meeting program objectives because in general, a belief in one’s own competency and ability to achieve goals is associated with improved success [11–13], and self-efficacy has been shown to improve retention of URM students in STEM [3, 14].

### Generalizability

There is much potential versatility for utilizing this Future Life Mapping framework. While our participants were all undergraduate students, this exercise can potentially be utilized at any life or career stage, from high school to mid-career. While we demonstrated the exercise was empowering and highly related among a URM cohort, we suspect this would be a valuable exercise across all demographics.

The objective of this exercise was not to inform participants of career options, but rather to empower participants with skills to consider more options and implement a strategy for making more informed career and life decisions. It was therefore encouraging to note that the two students who reported feeling less informed following the session also reported feeling more empowered. This further highlights the versatility of the exercise. Our session leader was an Assistant Professor in a STEM field; however, it is not necessary that session leaders have educational and career experience so closely aligned with participants’ interests. Session leaders need not be able to provide participants details and requirements of specific career and life trajectories to empower participants with career and life planning skills. Participants can also use the instructional materials provided and skills gained during the exercise with future life and career decisions unrelated to those they explored during the original exercise.

### Limitations

In this study we surveyed participants at the end of the exercise but did not collect follow up data to study the impact of the exercise on participant perceptions and future life and career planning. For this reason, we cannot draw definitive conclusions on the impact of this exercise on participants’ career and life trajectories, nor on long-term resiliency. We also were not able to determine if participants continued to use the future life map tool for planning and decision making moving forward, as the session leader encouraged throughout the exercise. While comments made by participants in the survey suggested possibly improved career planning self-efficacy, we did not utilize a validated tool to measure this as a part of our survey. These are all areas for potential future study.

## Conclusion

The Future Life Map exercise was successful in improving participant awareness of career options and helped learners to feel more empowered. Many became more aware of alternate routes and back-up plans that would allow them to meet career goals in the face of obstacles. While this exercise might be used with a wide variety of audiences, we are encouraged that this was a successful exercise in under-represented minority students relatively early in their STEM career journeys.

## Supporting information

S1 Appendix Future Life Map guidelines to be distributed to participants before the session to familiarize them with the concept and structure of the Future Life Map. (DOCX)

S2 Appendix Powerpoint presentation slides describing the importance of the exercise, and providing further instructions to participants in the creation of their own Future Life Maps. (PPTX)

S3 Appendix Primer questions that can be used to guide the discussion as necessary during session two. (DOCX)

S4 Appendix Post survey format. (DOCX)

S5 Appendix Supplementary data. (DOCX)
