# Supplementary material for: Use of the “Future Life Map” exercise to improve awareness of career options and opportunities in underrepresented minority undergraduate students pursuing STEM careers
Source: PLoS One. 2022 Feb 10;17(2):e0263848. doi: 10.1371/journal.pone.0263848 (PMC8830657; doi:10.1371/journal.pone.0263848)
Supplement: S1 Appendix — (DOCX) [file pone.0263848.s001.docx]

**“The Future Life Map”**

**Goal:**

To create a detailed map of your potential future opportunities starting from where you currently sit.

**Introduction:**

This is a bit different than perhaps most life maps in that the point of this map is not to map out how to get to a single specific future goal but rather to map out all reasonable options that you have based on decisions that you make as you go through life (a *what if?* life thought experiment). Similar to how a street map doesn’t just show you the road you are on or the path to get to a single destination but rather it shows all of the side streets that you could potentially turn on as you go. Even GPS mapping systems display cross streets and alternate route options. Some of these roads lead you to different destinations while others might get you to the same destination but take a bit longer. Sometimes there are detours due to construction (like SARS-CoV-2) on a path and other times you might specifically decide to take a detour to stop at a scenic lookout on the trip.

At every intersection, a decision is made to continue onward or change direction. Sometimes these decisions are seemingly insignificant while other times small decisions can have long lasting effects on your path. These decisions are sometimes informed decisions (perhaps you are following a route that many have taken previously) while other decisions or paths are less clear. Moreover, sometimes decisions are made based on preconception which may or may not be rooted in truth. When a choice is made to take a specific road, it means that you are choosing to not take any of the other roads at that intersection. This exercise will help you map out various intersection decision points on your life map and will provide you an opportunity to assess what each path would mean for you if you elected to go that route.

By the end of this exercise you will have a comprehensive, and personal, map of future options with a much more in depth understanding of opportunities. This will help you then focus and narrow your goals to achieve a specific outcome all the while understanding what other opportunities you are choosing not to take.

**Guidelines (read all directions before starting the “Future Life Map”):**

1. The map should look like a web starting from where you currently are in life. For all of you this means currently being enrolled in an undergraduate program.
   1. Take a large sheet of paper (poster board optional) and circle “Undergrad” at the bottom middle of the page. While the example below is digital I highly recommend you perform this task on pen and paper.
2. From this starting point draw branches from it to connect to decision points / opportunities / prospects… An example of this would be “Undergrad” linking to a “Job” and “Grad School” and so on. All options that you can think of **(good, bad, of interest, and not of interest)** should be mapped. These options will likely not be the same for every individual. Mapping all options is critical as it is just as important to have information ruling a specific path in as it is to have the necessary information to ensure you are appropriately ruling something out.
   1. Some of these decision points can be self-imposed (i.e. deciding not to apply to grad school) vs imposed upon you (i.e. applying to grad school but not getting accepted).
   2. Mapping alternative options now will help prepare you for unforeseen life events in the future.
3. At each point you should put some details about what you can expect to achieve (or what your life would be like) at that point or what new opportunities that achieving that point affords you for next steps.
   1. Use green ink to designate *benefits* and red ink to designate *drawbacks* at each decision point.
   2. An example of this at the “Job” post “Undergrad” location on the life map might include: anticipated salary with your degree, benefits package, type of job you could reasonably expect to get with your current credentials. A job outlook circle might stem as an additional link to the “Job” circle depending upon the type of job opportunities that exist in your field.
   3. Once you fill out these brief details you should take a moment to consider if you would be happy in that role or not. This exercise will help you focus your efforts on paths that you feel are of more interest to you while being aware of all other potential avenues.
4. You should also include details regarding the demands to achieve each next potential step. For example…if you want to go to medical school you should have an idea/include details regarding average GPA, or competitiveness of a subspecialty that you are interested in. If you are unclear of the requirements needed to achieve a specific next step then now is a good time to do some research. This includes learning about standard time-lines since many graduate programs and even jobs have specific hiring and application cycles and pre-requisites that you need to be aware of. Missing a prerequisite or hiring cycle deadline might delay you an entire year from achieving the next step/goal on your life map.
5. This life map will be very detailed at times and very broad at times. The exercise is intended to give you an opportunity to formally think through different options on paper. This will help you validate or refute preconceptions about career and school paths that maybe you have not taken enough time to fully understand/research until now.
6. This map should be carried out through a number of transitions and depending on the types of career paths ~10-20 yrs out from the current date is typical.
7. **Additional options:**
   1. Up until this point the life map focus has been primarily centered on the individual but life tends not to be that simple. If you would like to add details regarding family/personal life that can also prove to be very useful as not all decisions are purely founded in education/work opportunities.
   2. Consider your return on investment (ROI). ROI can be in terms of time as well as money. For example: an additional degree may result in a higher salary but considering the time or debt associated to attain that degree may reduce your overall ROI. This is not to say that long school paths or high levels of debt to achieve specific goals should be avoided but rather you should be aware of what those time frames and dollar amounts typically look like.


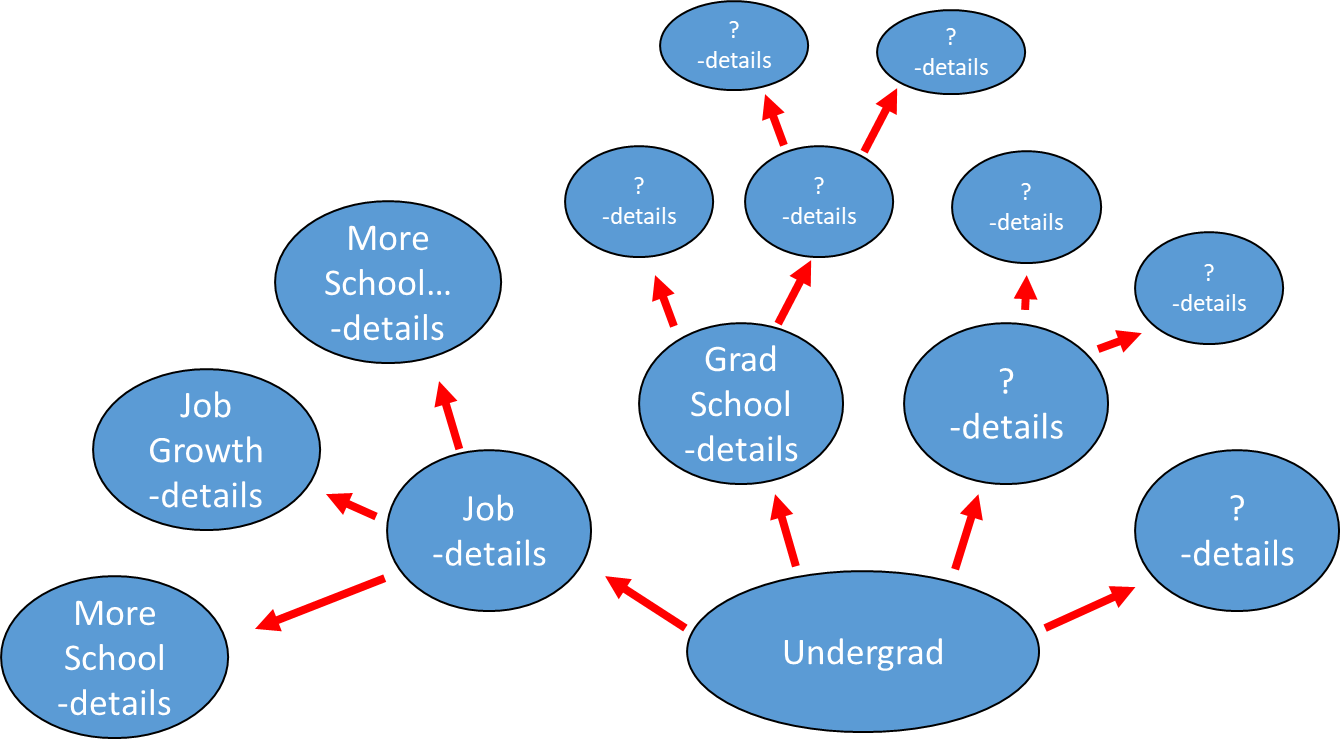


**Figure 1:** “Future Life Map” Example Template

**Conclusions:**

Life is complicated and there are many points at which decisions are made that affect future potential opportunities. Some aspects of the life map are intended to help you think through difficult decisions or to better prepare you for the path that you would like to pursue. The goal of the life map is not intended to discourage any specific path but rather to give a realistic view of the potential hurdles necessary to achieve success on a specific path. Revisiting the life map and reworking opportunities as you progress through the various arms of the life map over time is advisable. Similar to roads in real life, sometimes new routes are created, roads become paved, construction projects end, and highways are widened to facilitate new opportunities.

*****While the life maps will be checked for assignment completion these life maps will not be collected and there will not be a requirement to share every detail that is included.

**Bonus Note:**

While the life map as presented here is intended to walk you through the process of creating a comprehensive life road map this same process can be utilized to assess single decision points in greater detail as well. Examples of utilizing this thought experiment technique in this manner would be: whether or not to take a specific job opportunity, attend a specific school, pick a specific major, buy a house or rent, purchase a specific car, and so on… Whether you consciously realize it or not, you are constantly making decisions and these decisions can affect your future opportunities.

**Tips for success in building your map:**

- Use the internet to identify job postings and program descriptions that are similar to those you are considering. These will likely list pre-requisites and give you a sense of what is needed for successful applications to these types of jobs and/or programs
- Spend some time doing a broad internet search to identify potential paths and opportunities that might not be immediately obvious to you. Terms might include: “"what jobs can a person with xx undergraduate degree get?" “What are unusual jobs in xx field?” These may seem like basic questions that you think you know the answer to, but the point is to broaden your awareness of the possibilities
- Reach out to your existing network (family, friends, other students, professors, and mentors) to gain from their experience and expertise with the different options you are considering
