# Supplementary material for: Use of the “Future Life Map” exercise to improve awareness of career options and opportunities in underrepresented minority undergraduate students pursuing STEM careers
Source: PLoS One. 2022 Feb 10;17(2):e0263848. doi: 10.1371/journal.pone.0263848 (PMC8830657; doi:10.1371/journal.pone.0263848)
Supplement: S2 Appendix — (PPTX) [file pone.0263848.s002.pptx]

## Slide 1
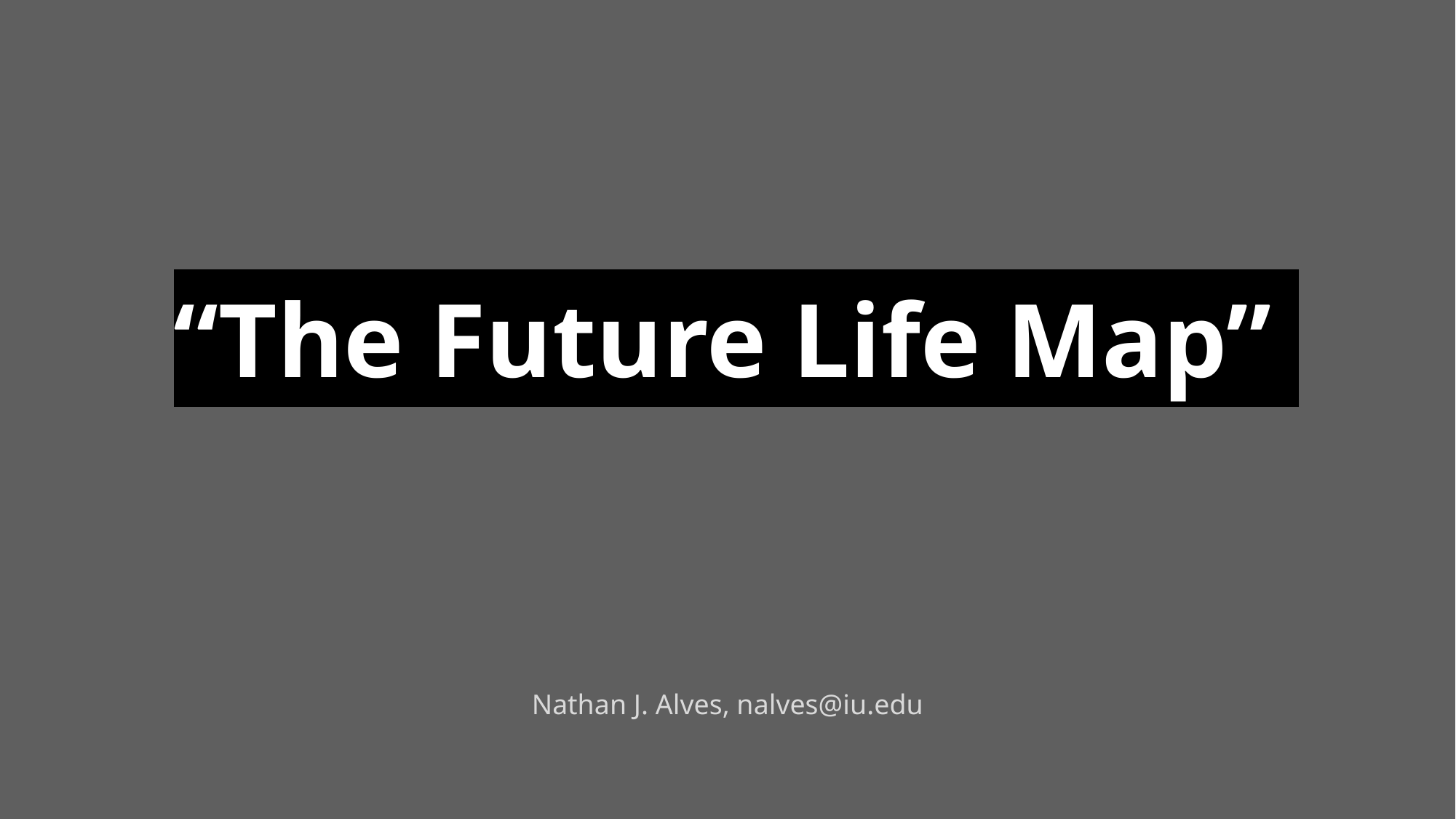

# “The Future Life Map”
Nathan J. Alves, nalves@iu.edu

## Slide 2
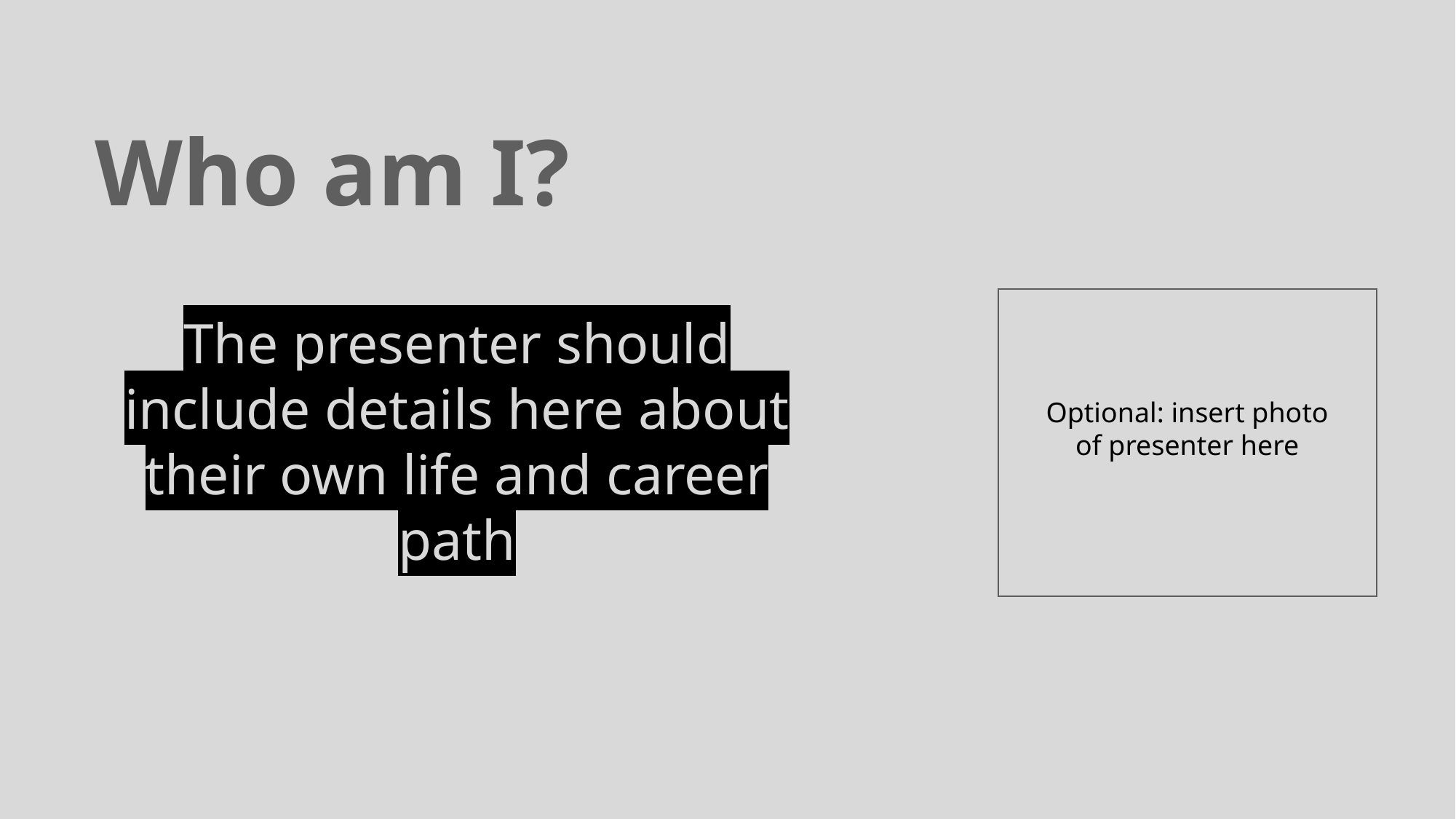

# Who am I?
The presenter should include details here about their own life and career path
Optional: insert photo of presenter here

## Slide 3
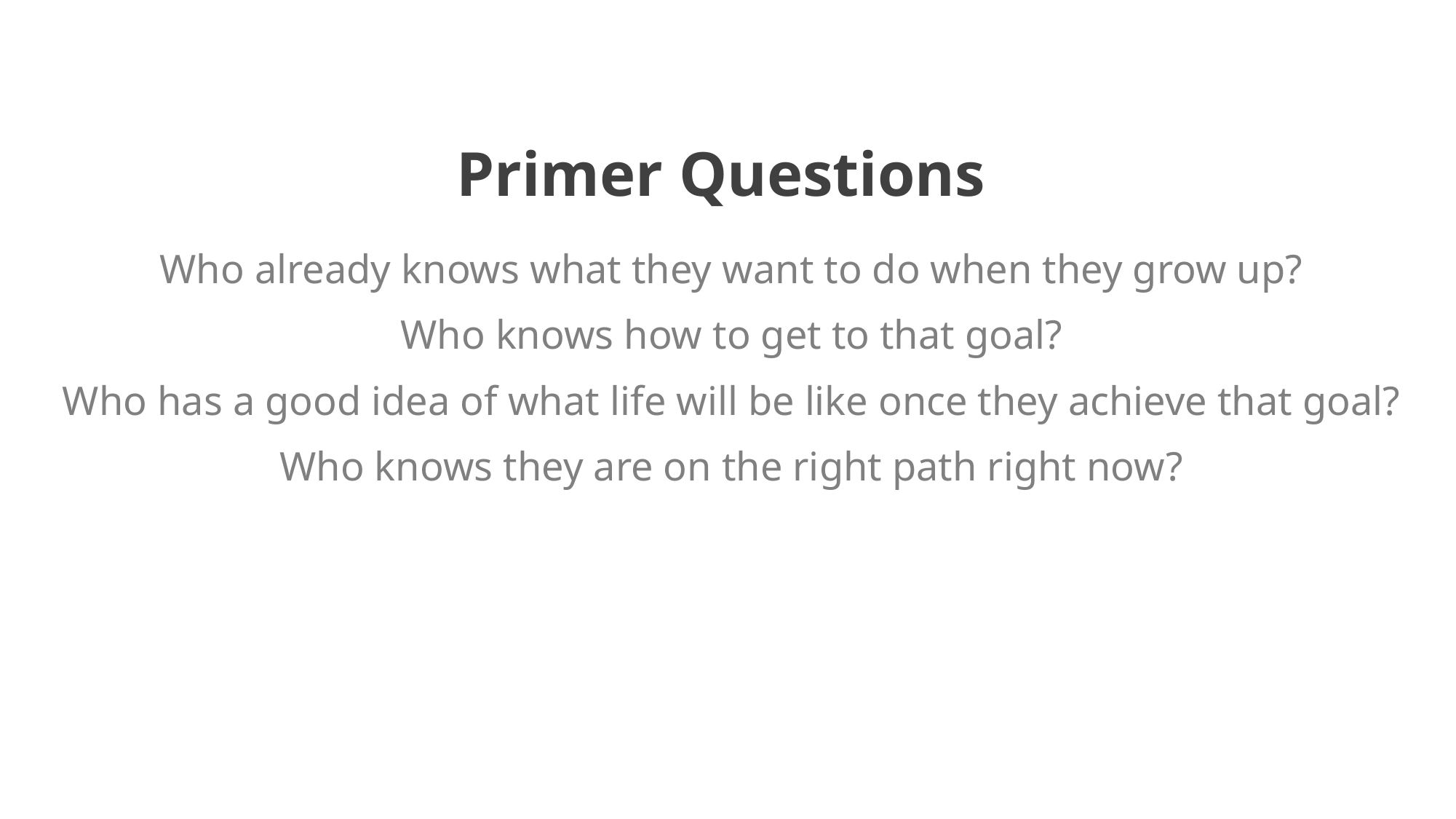

# Primer Questions
Who already knows what they want to do when they grow up?
Who knows how to get to that goal?
Who has a good idea of what life will be like once they achieve that goal?
Who knows they are on the right path right now?

## Slide 4
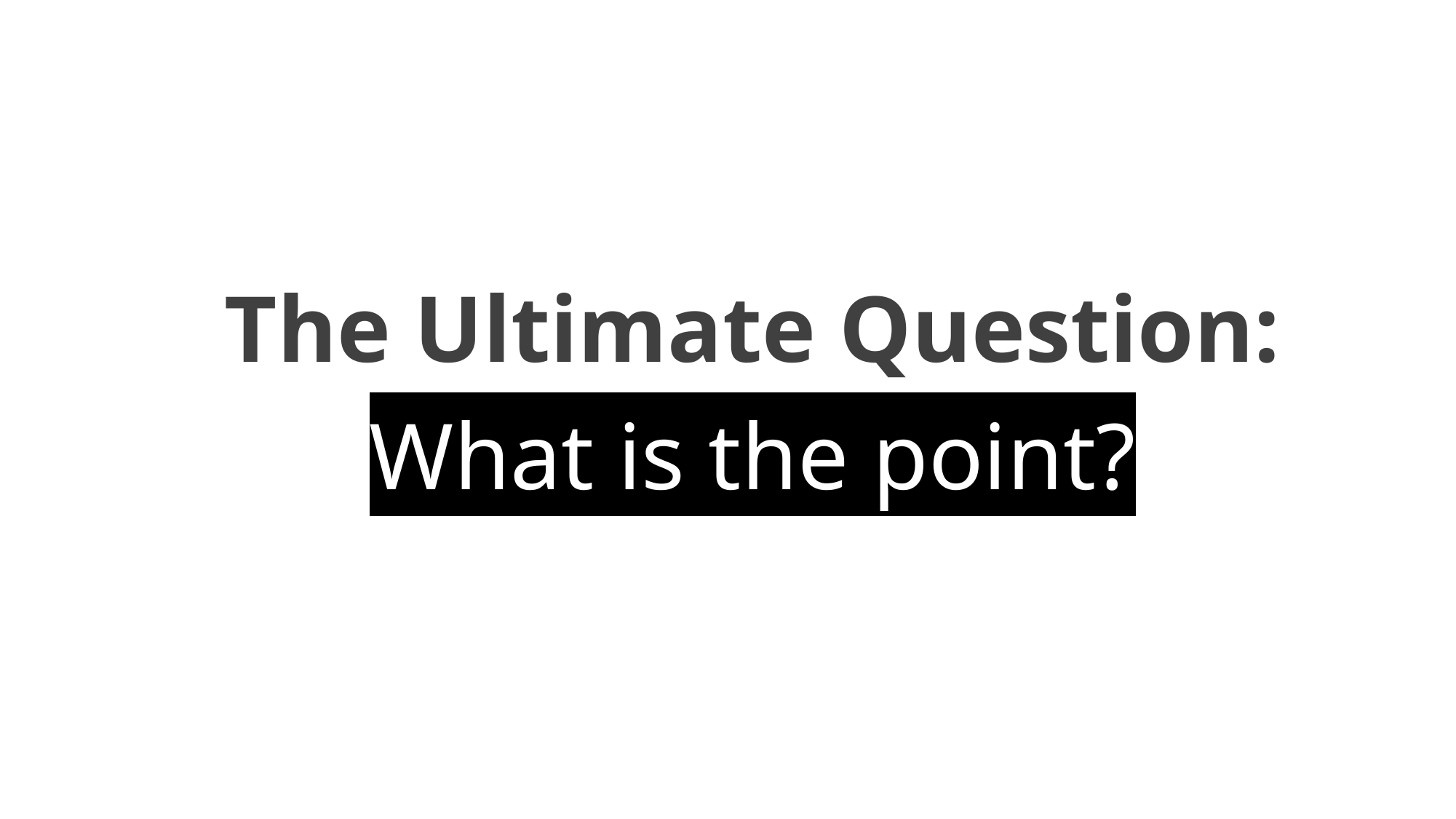

The Ultimate Question:
What is the point?

## Slide 5
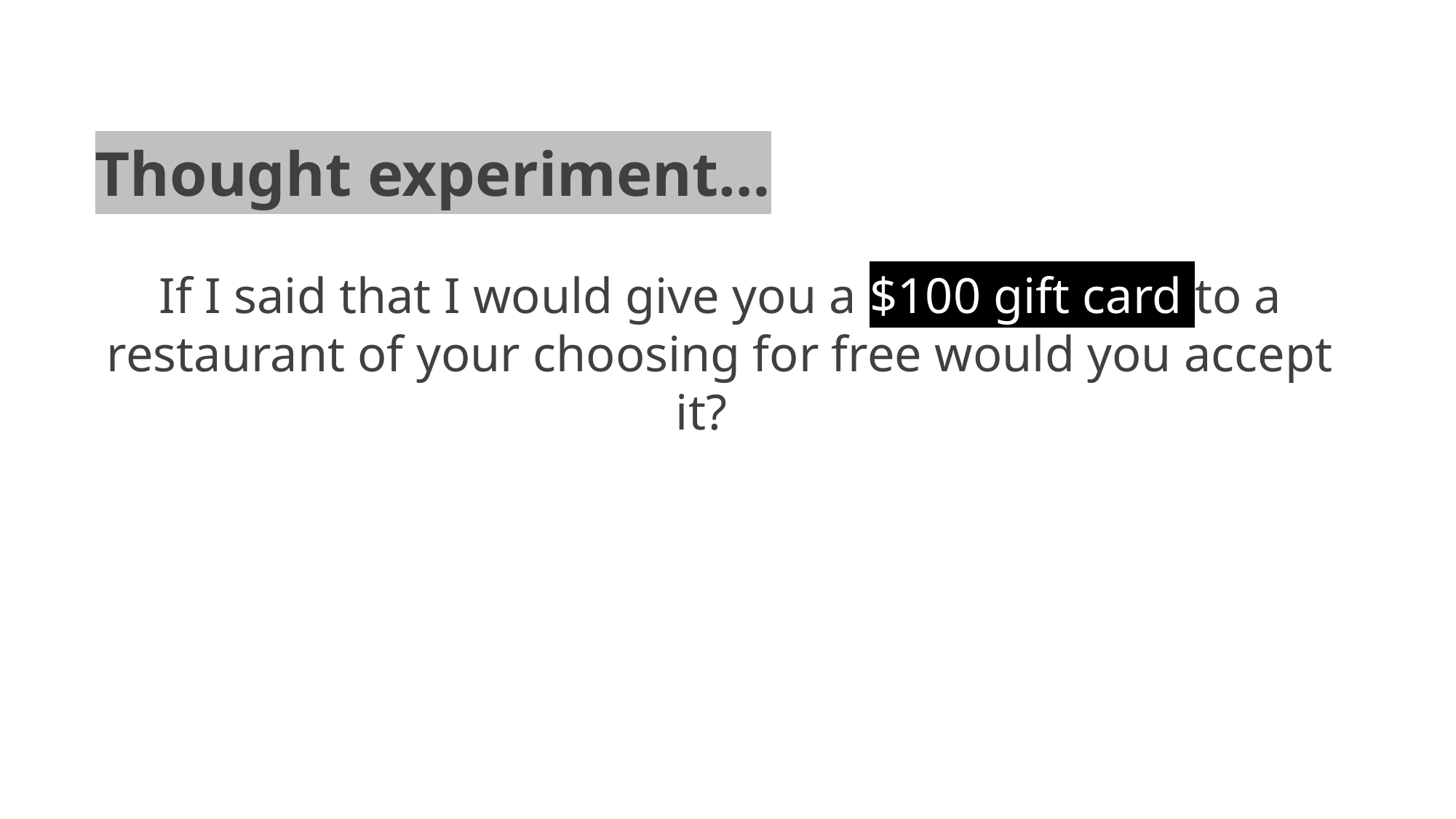

# Thought experiment…
If I said that I would give you a $100 gift card to a restaurant of your choosing for free would you accept it?

## Slide 6
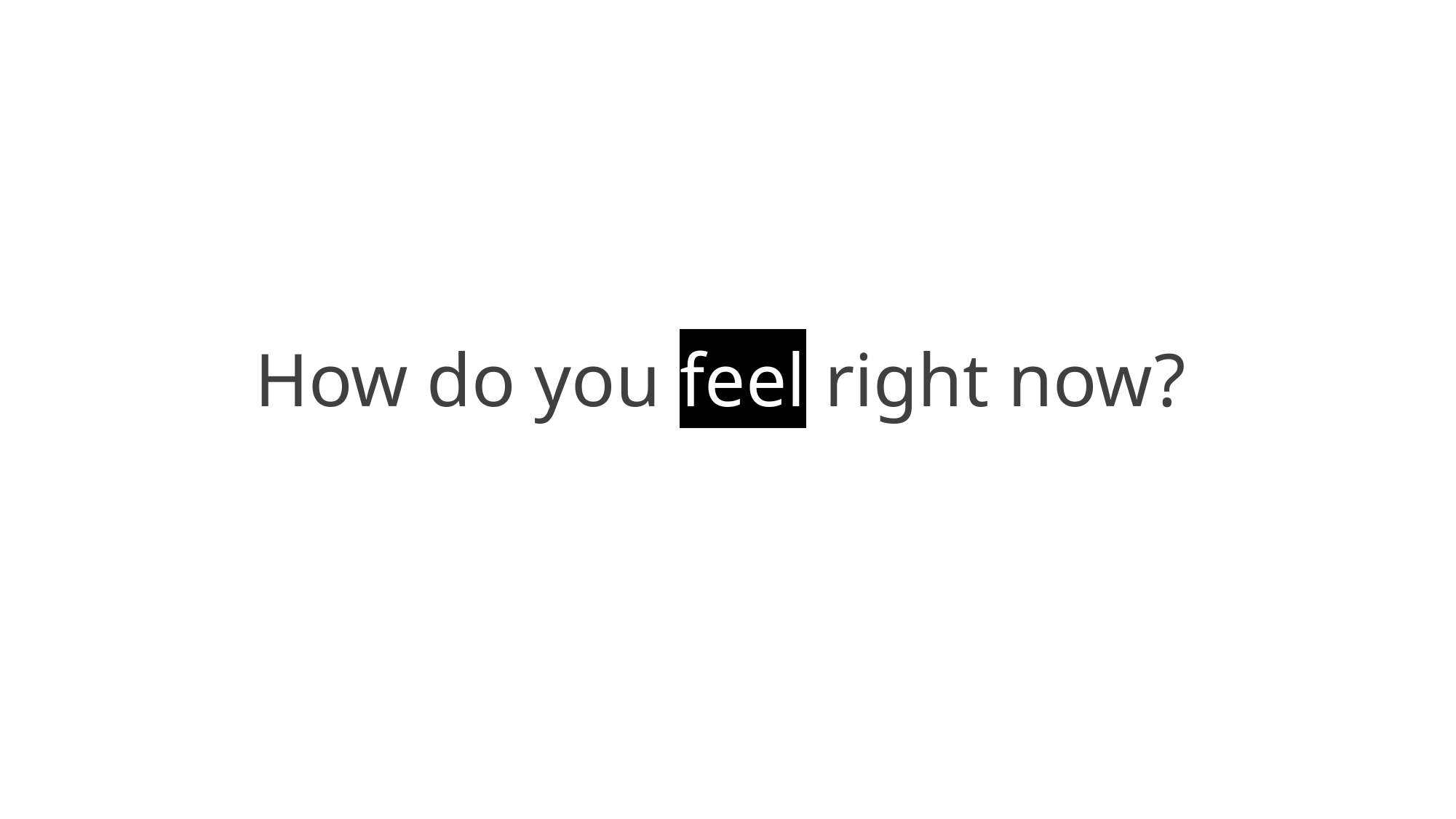

How do you feel right now?

## Slide 7
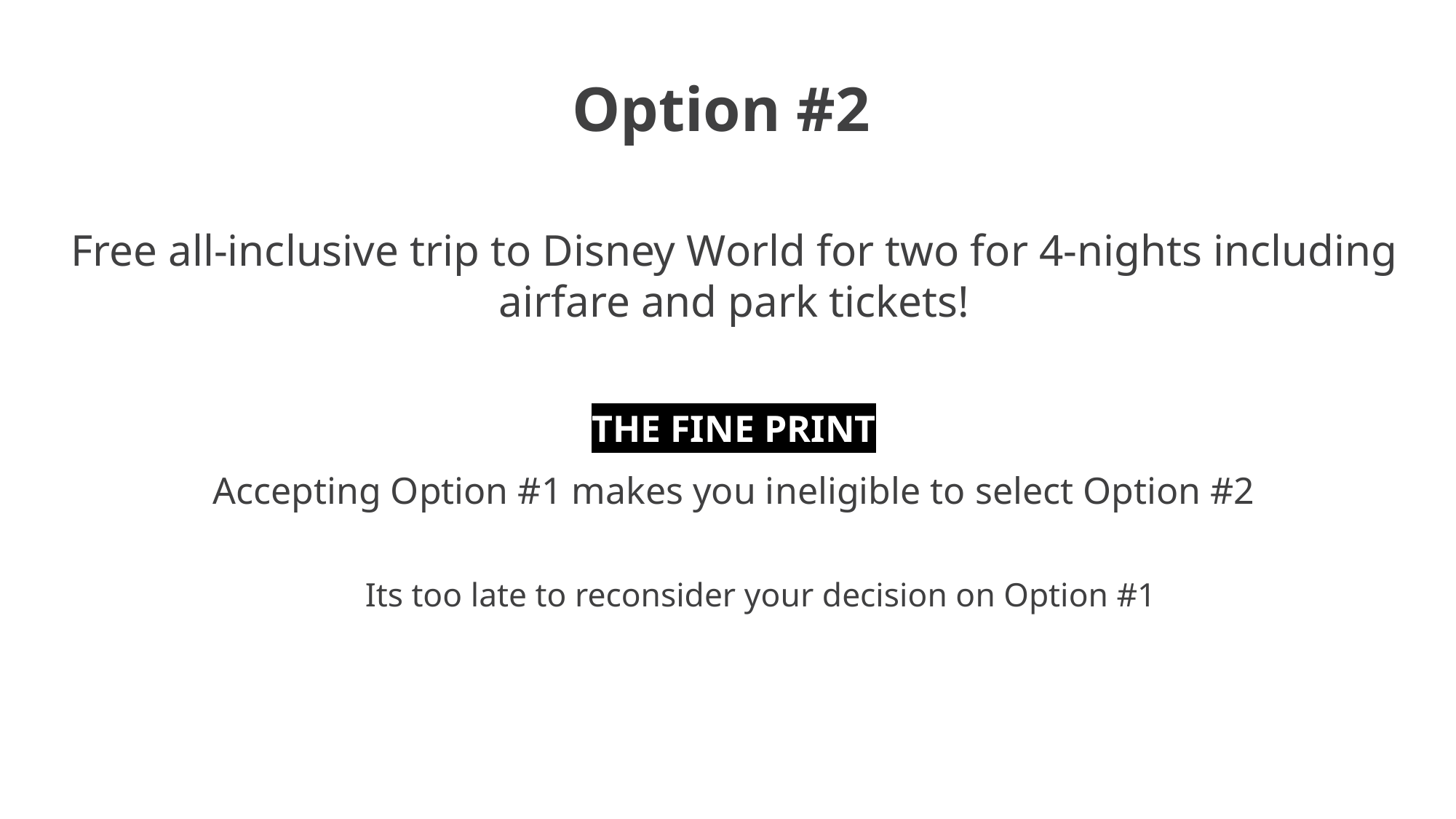

# Option #2
Free all-inclusive trip to Disney World for two for 4-nights including airfare and park tickets!
THE FINE PRINT
Accepting Option #1 makes you ineligible to select Option #2
Its too late to reconsider your decision on Option #1

## Slide 8
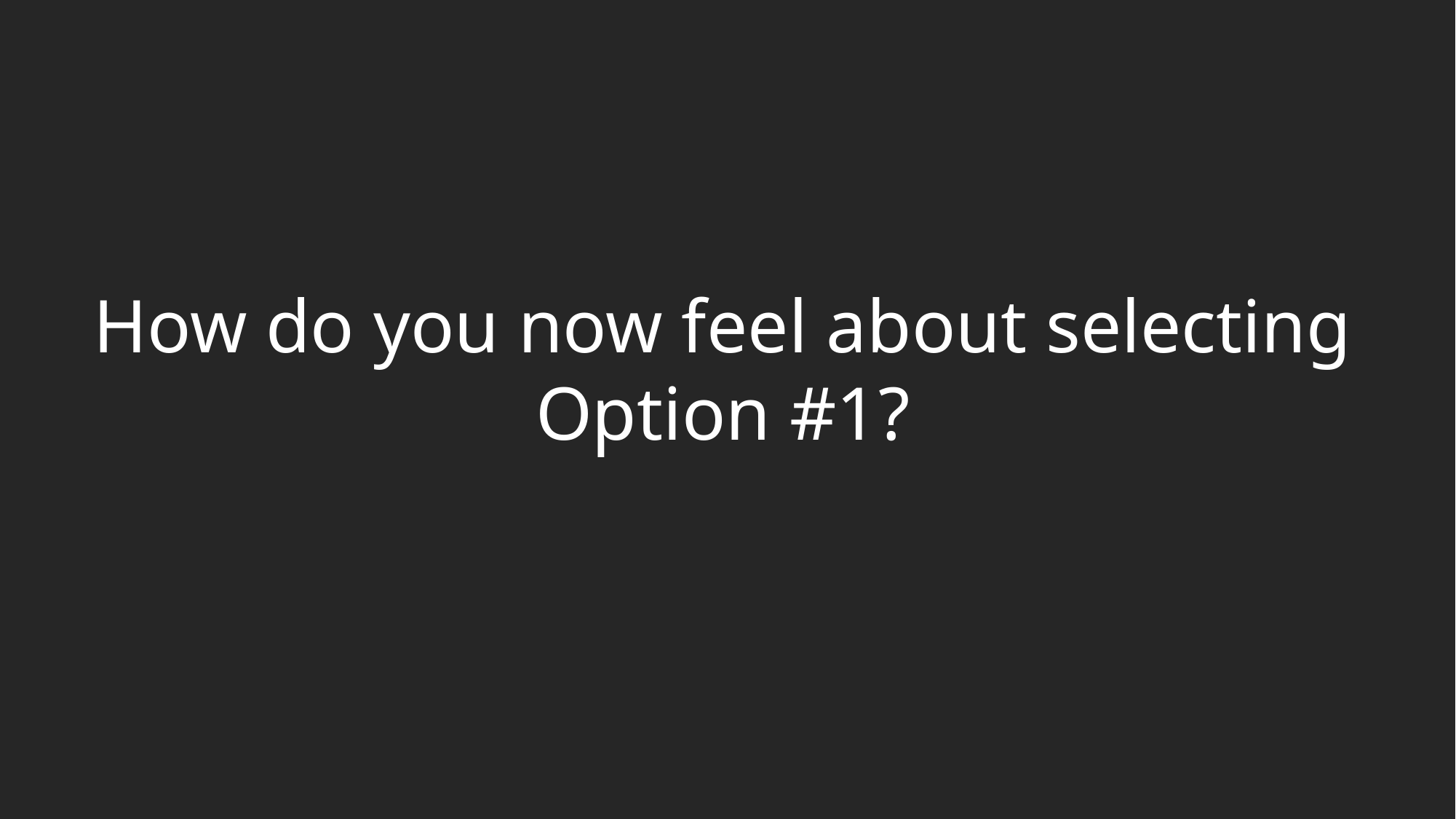

How do you now feel about selecting Option #1?

## Slide 9
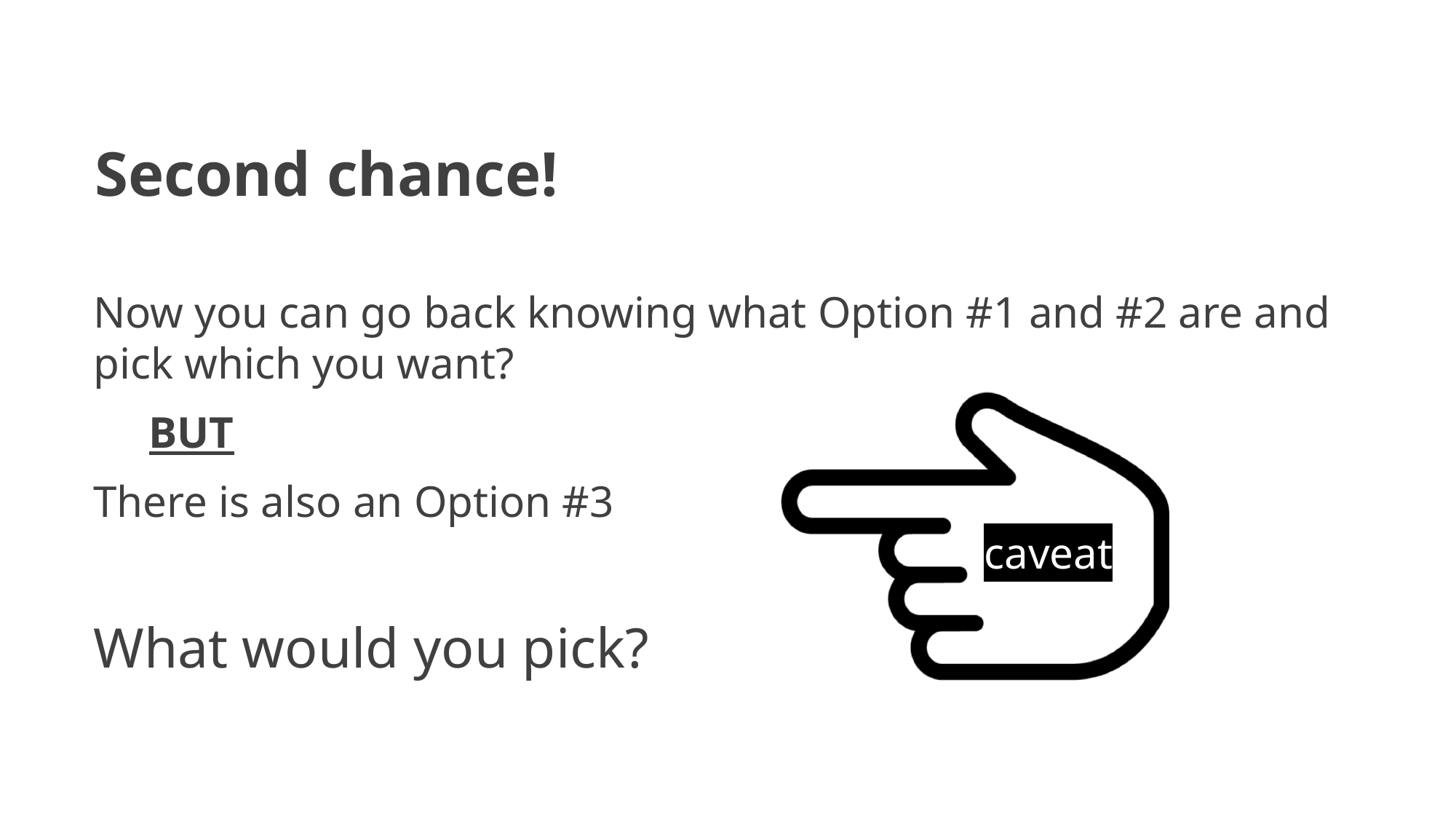

# Second chance!
Now you can go back knowing what Option #1 and #2 are and pick which you want?
 BUT
There is also an Option #3
What would you pick?
caveat

## Slide 10
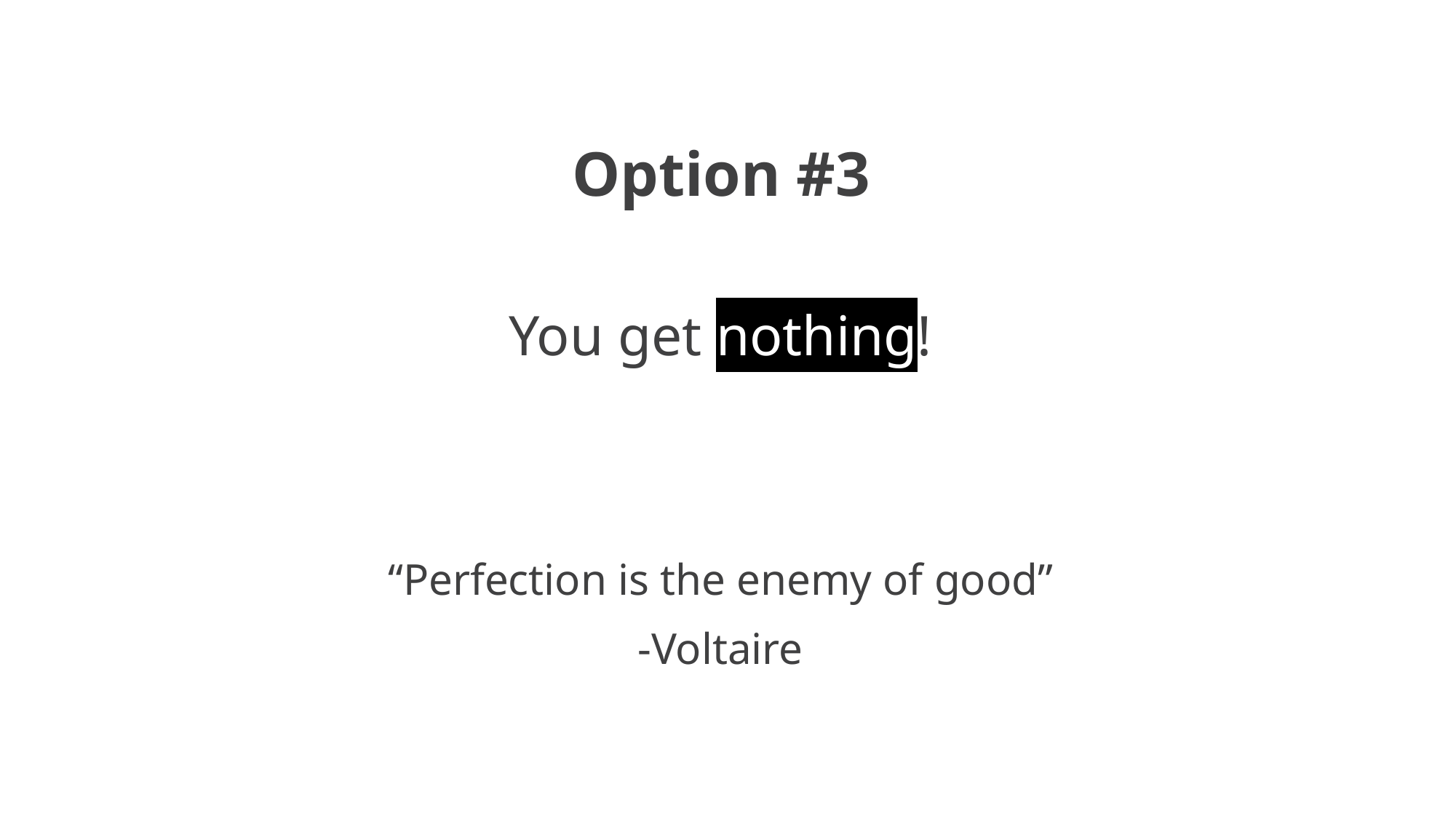

# Option #3
You get nothing!
“Perfection is the enemy of good”
-Voltaire

## Slide 11
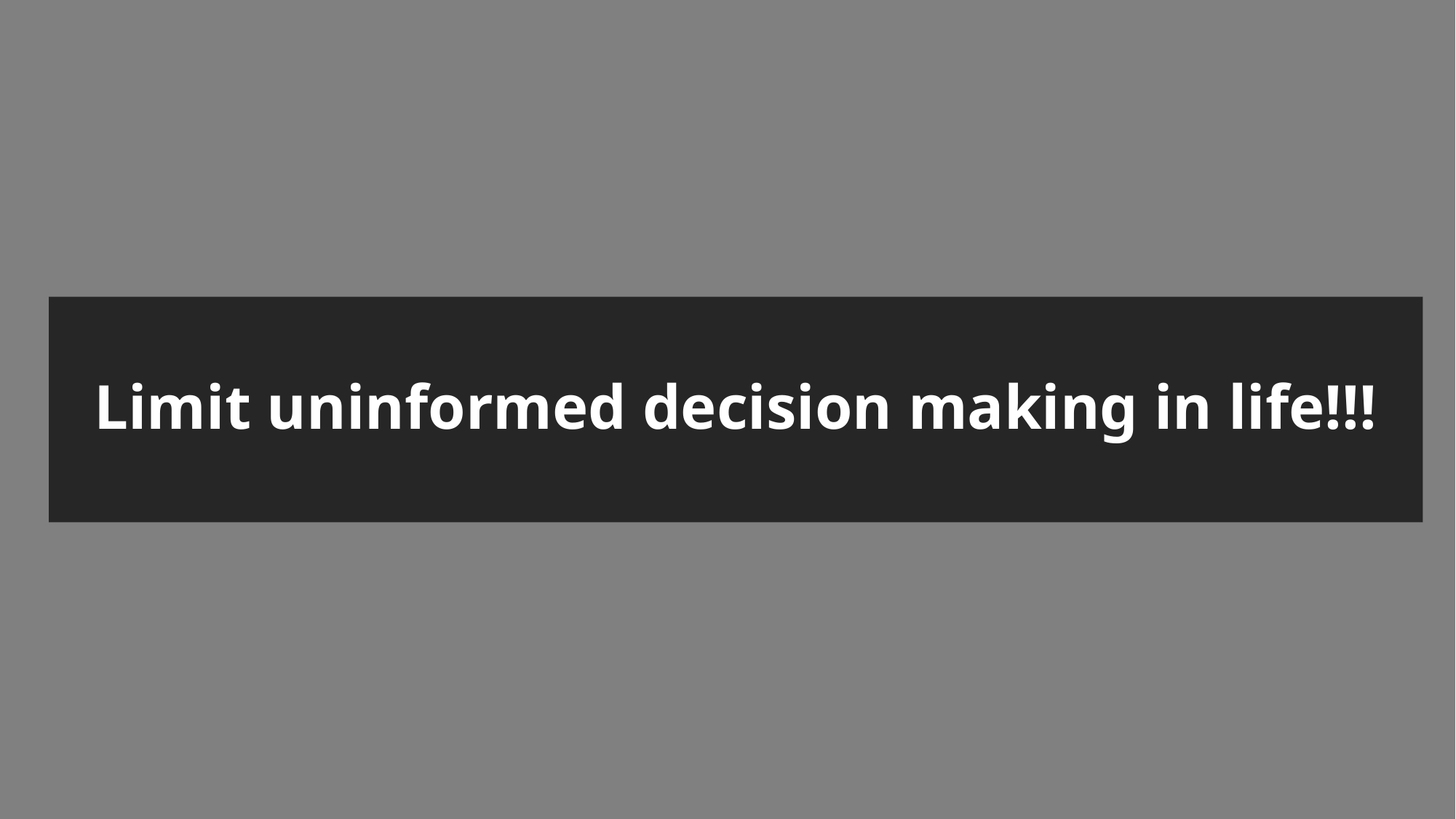

# Limit uninformed decision making in life!!!

## Slide 12
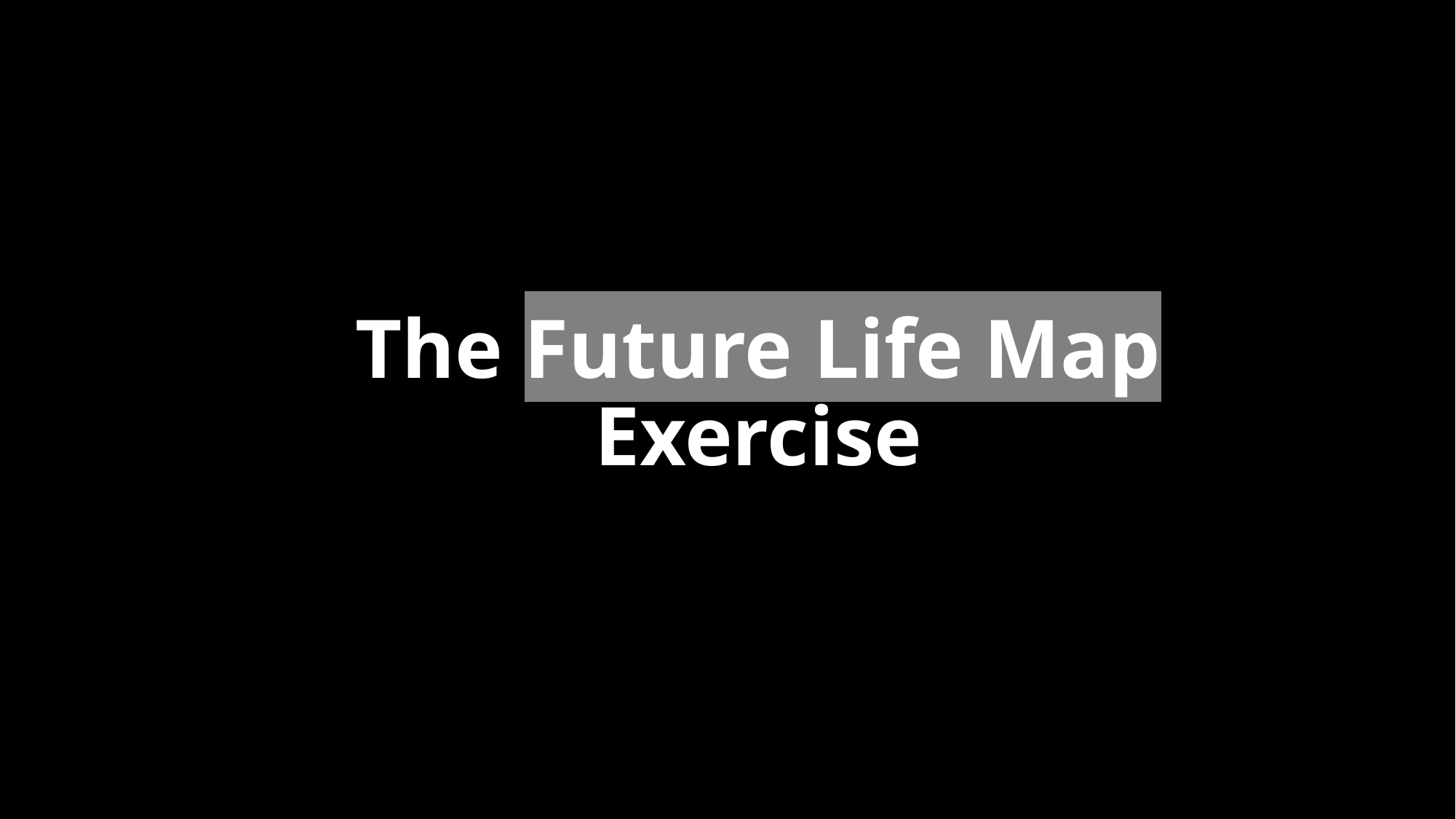

# The Future Life Map Exercise

## Slide 13
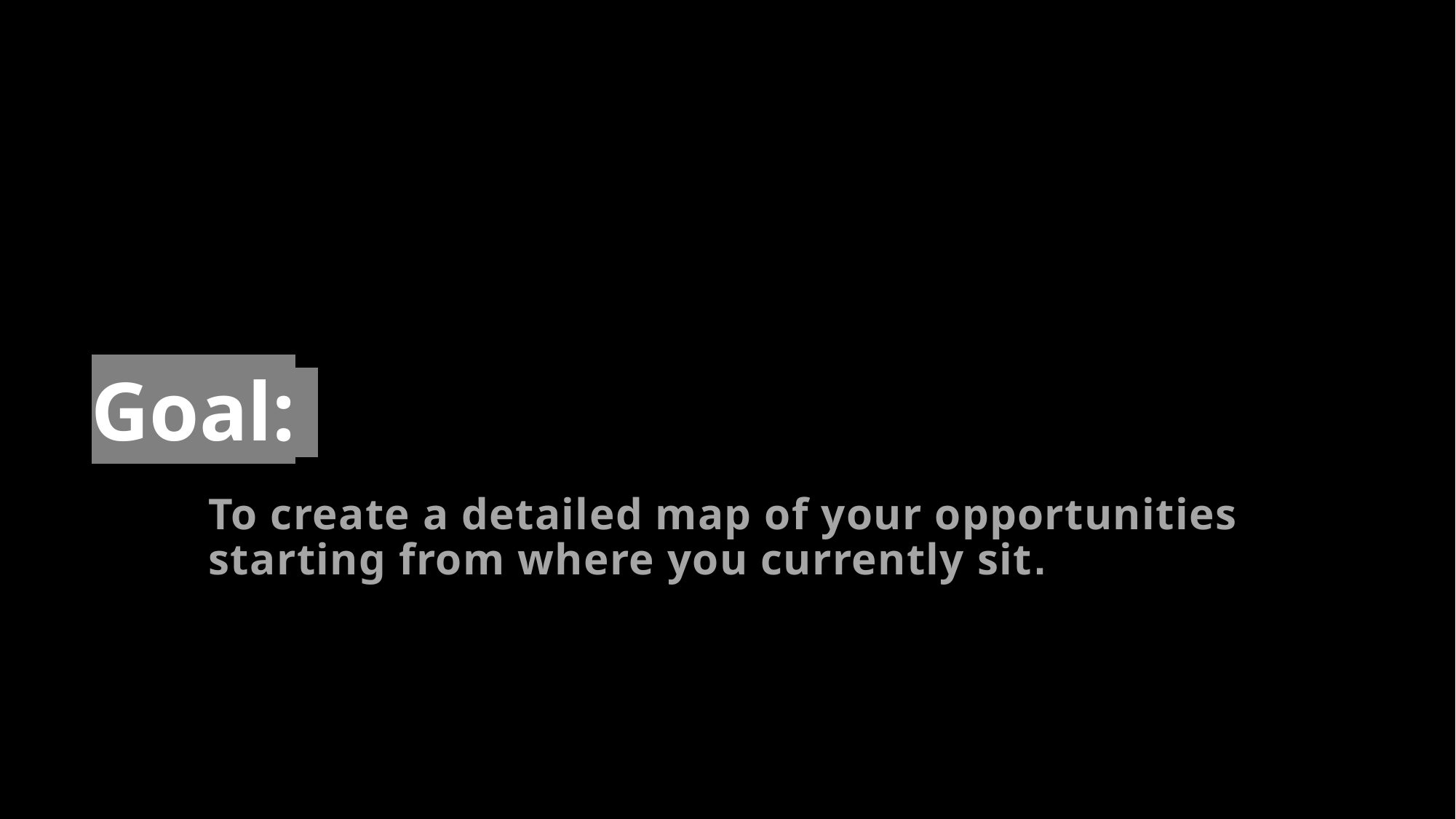

# Goal:
To create a detailed map of your opportunities starting from where you currently sit.

## Slide 14
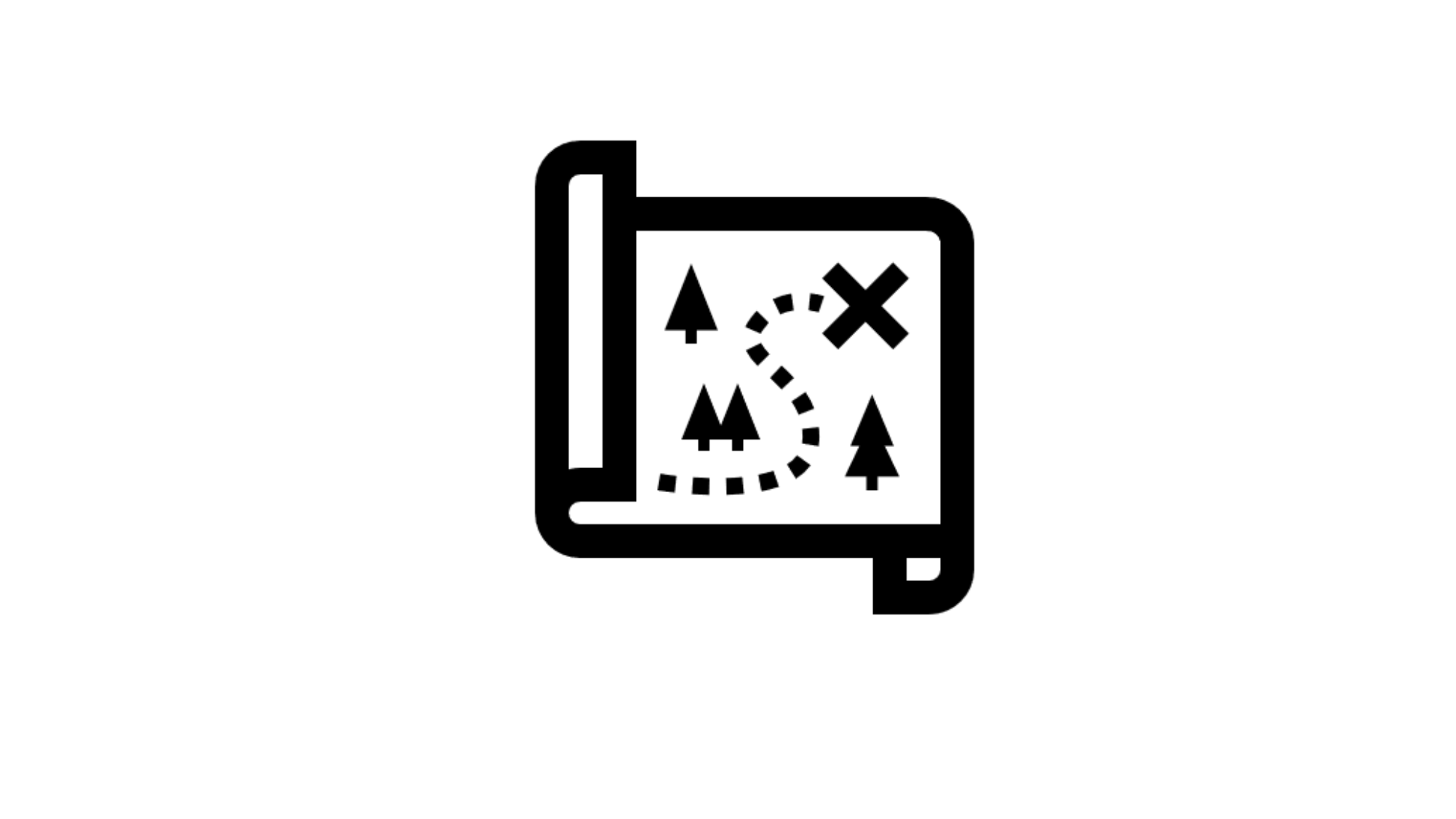

## Slide 15
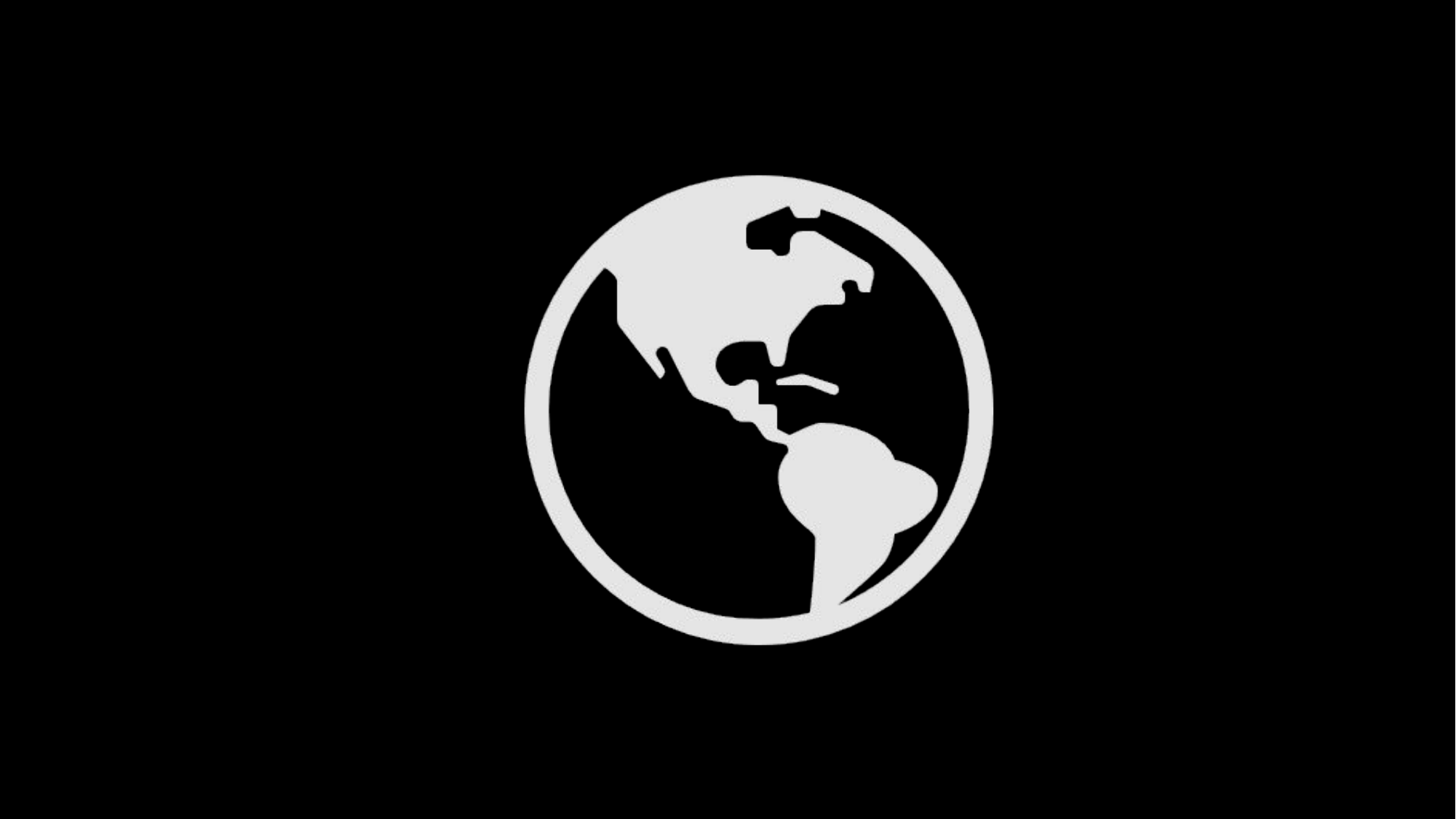

## Slide 16
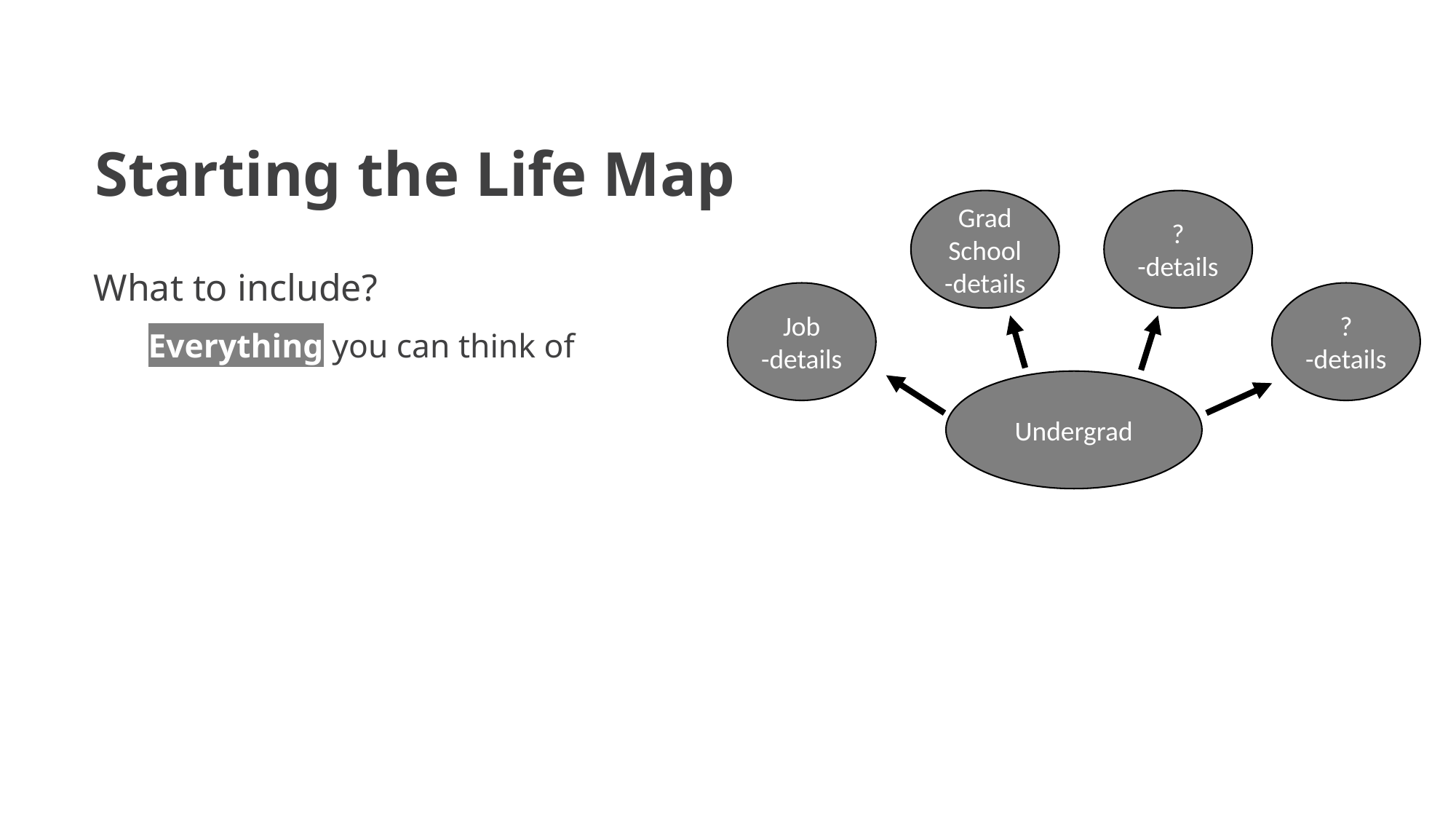

# Starting the Life Map
Grad School
-details
?
-details
What to include?
Everything you can think of
Job
-details
?
-details
Undergrad

## Slide 17
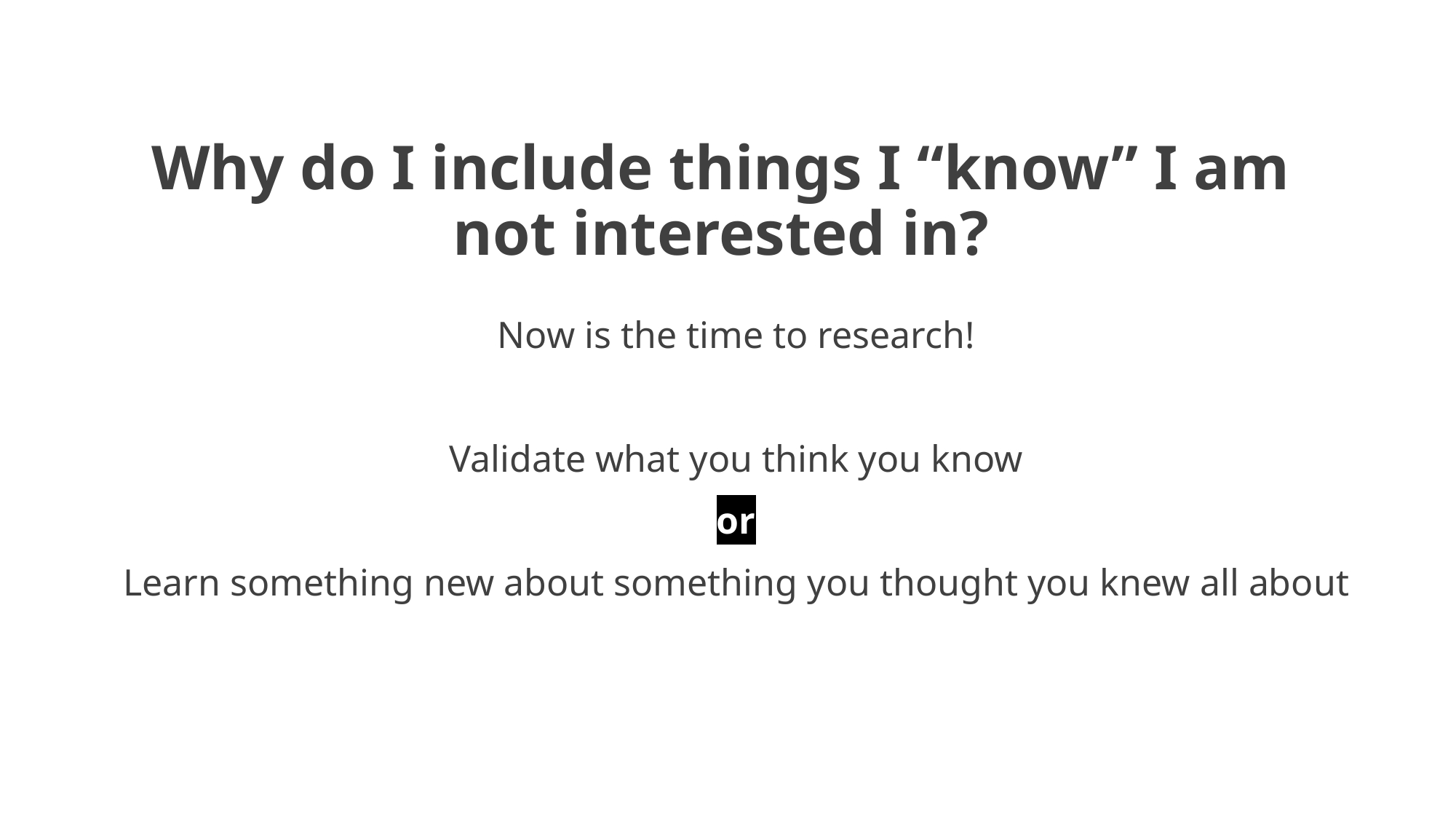

# Why do I include things I “know” I am not interested in?
Now is the time to research!
Validate what you think you know
or
Learn something new about something you thought you knew all about

## Slide 18
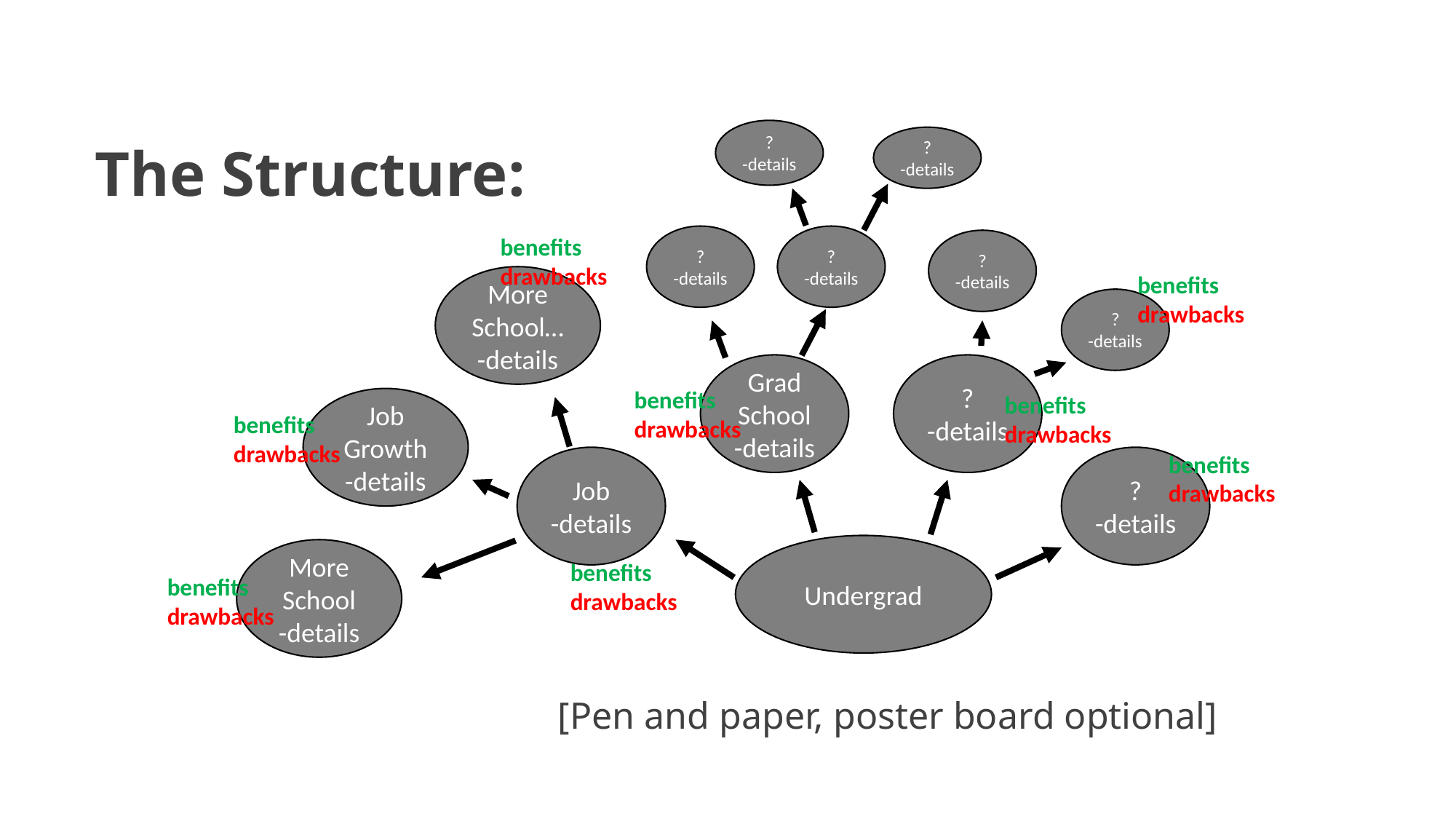

?
-details
# The Structure:
?
-details
benefits
drawbacks
?
-details
?
-details
?
-details
benefits
drawbacks
More School…
-details
?
-details
Grad School
-details
?
-details
benefits
drawbacks
benefits
drawbacks
Job Growth
-details
benefits
drawbacks
benefits
drawbacks
Job
-details
?
-details
Undergrad
More School
-details
benefits
drawbacks
benefits
drawbacks
[Pen and paper, poster board optional]

## Slide 19
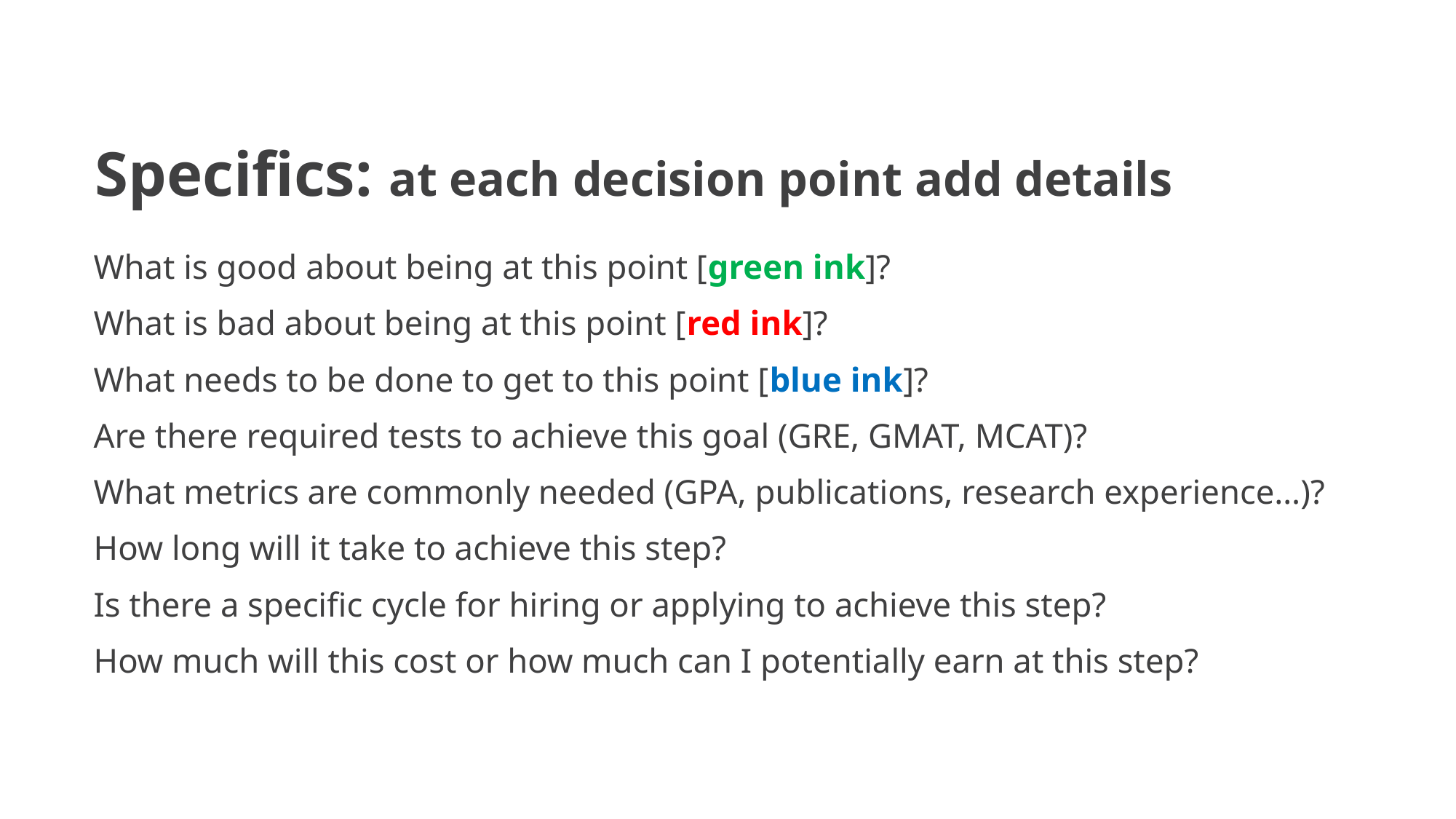

# Specifics: at each decision point add details
What is good about being at this point [green ink]?
What is bad about being at this point [red ink]?
What needs to be done to get to this point [blue ink]?
Are there required tests to achieve this goal (GRE, GMAT, MCAT)?
What metrics are commonly needed (GPA, publications, research experience…)?
How long will it take to achieve this step?
Is there a specific cycle for hiring or applying to achieve this step?
How much will this cost or how much can I potentially earn at this step?

## Slide 20
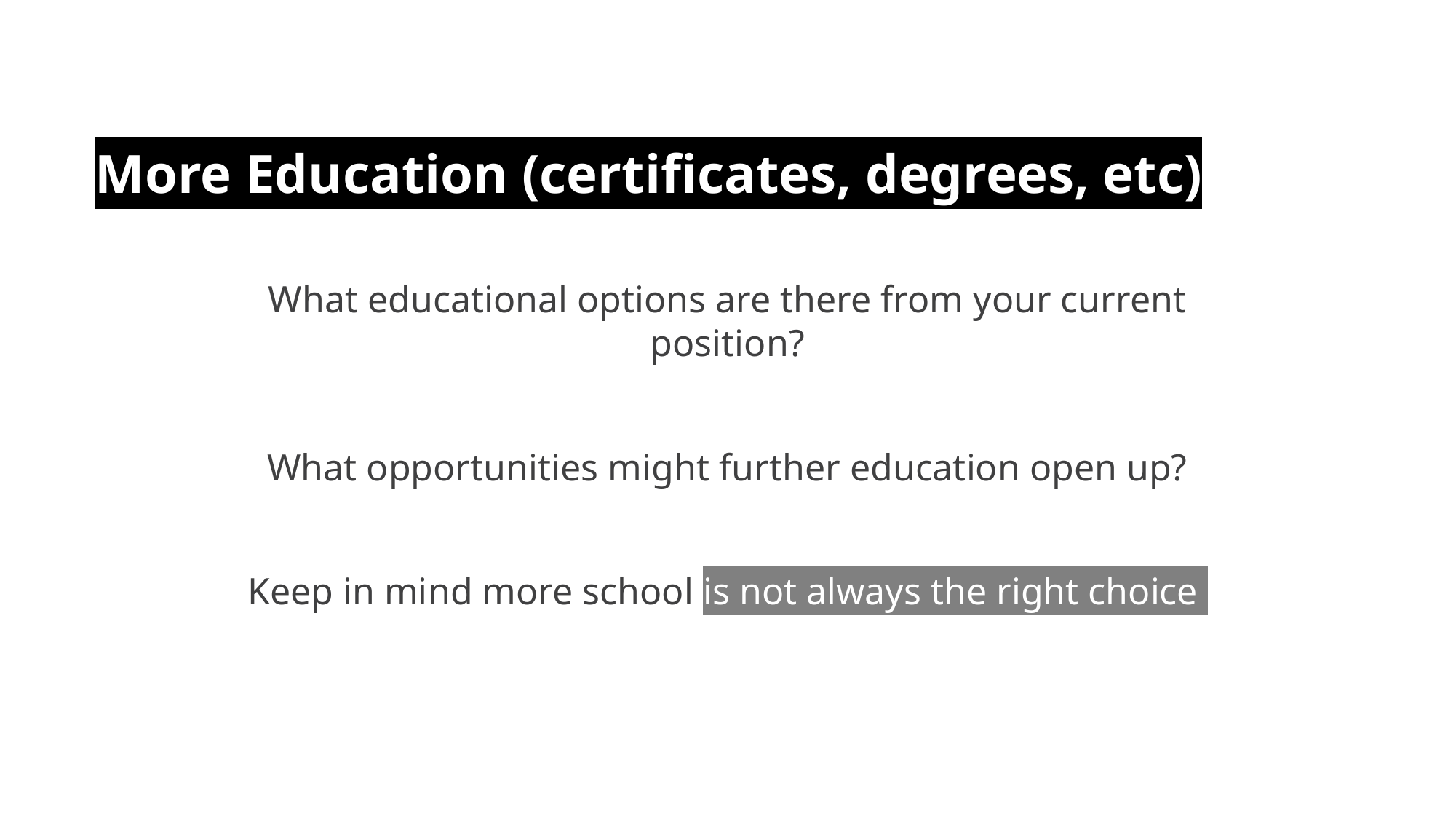

# More Education (certificates, degrees, etc))
What educational options are there from your current position?
What opportunities might further education open up?
Keep in mind more school is not always the right choice

## Slide 21
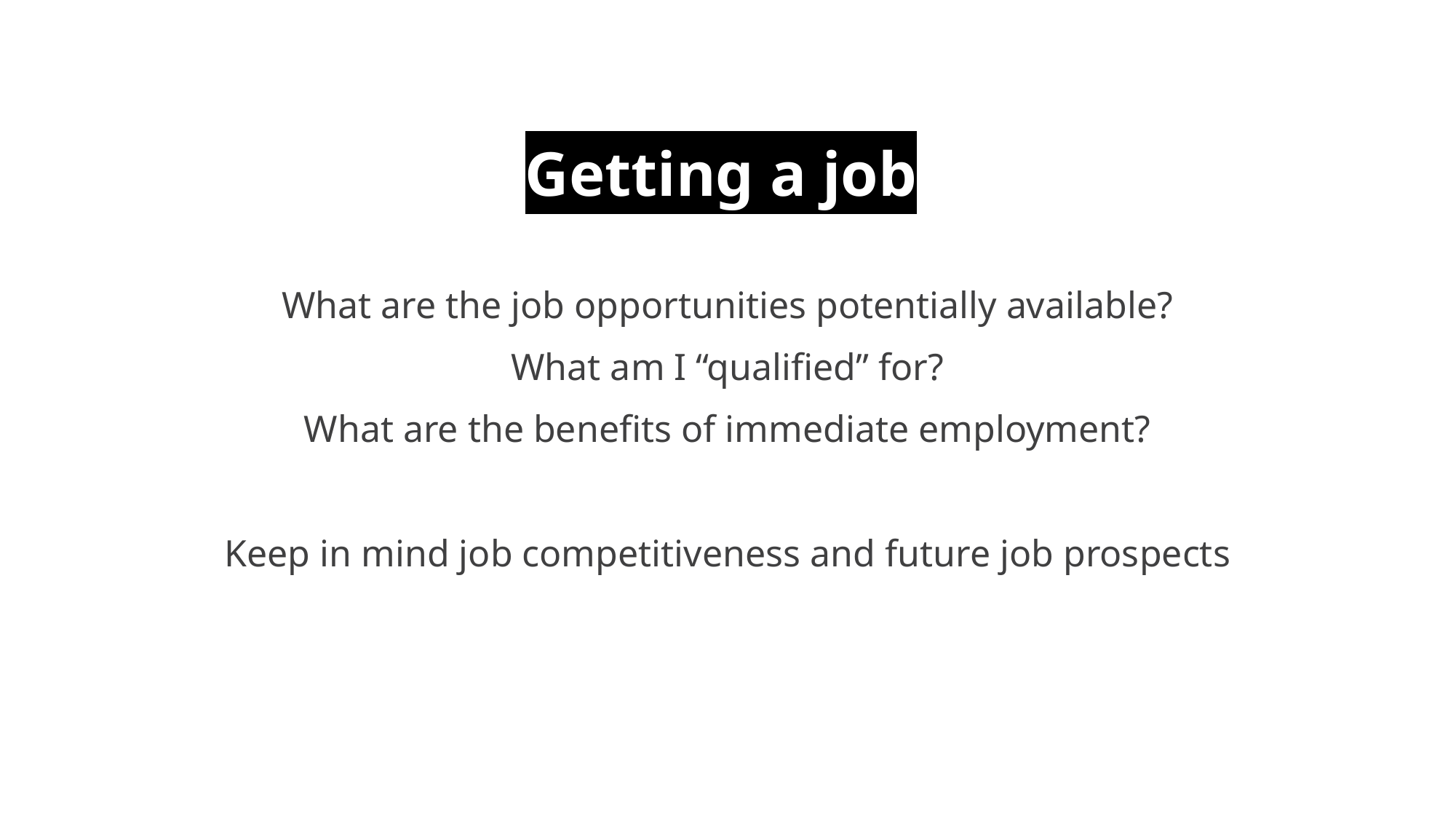

# Getting a job
What are the job opportunities potentially available?
What am I “qualified” for?
What are the benefits of immediate employment?
Keep in mind job competitiveness and future job prospects

## Slide 22
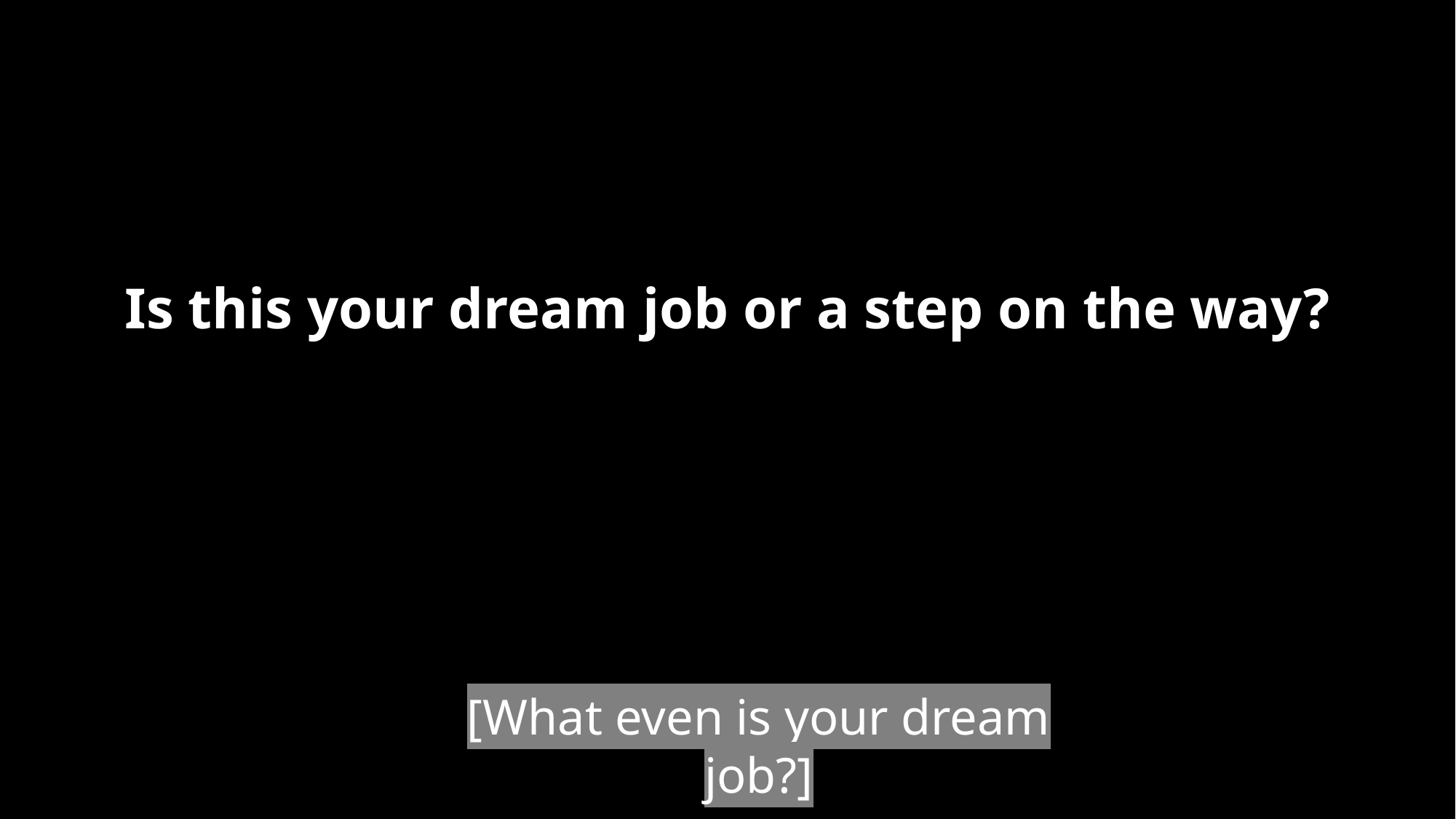

# Is this your dream job or a step on the way?
[What even is your dream job?]

## Slide 23
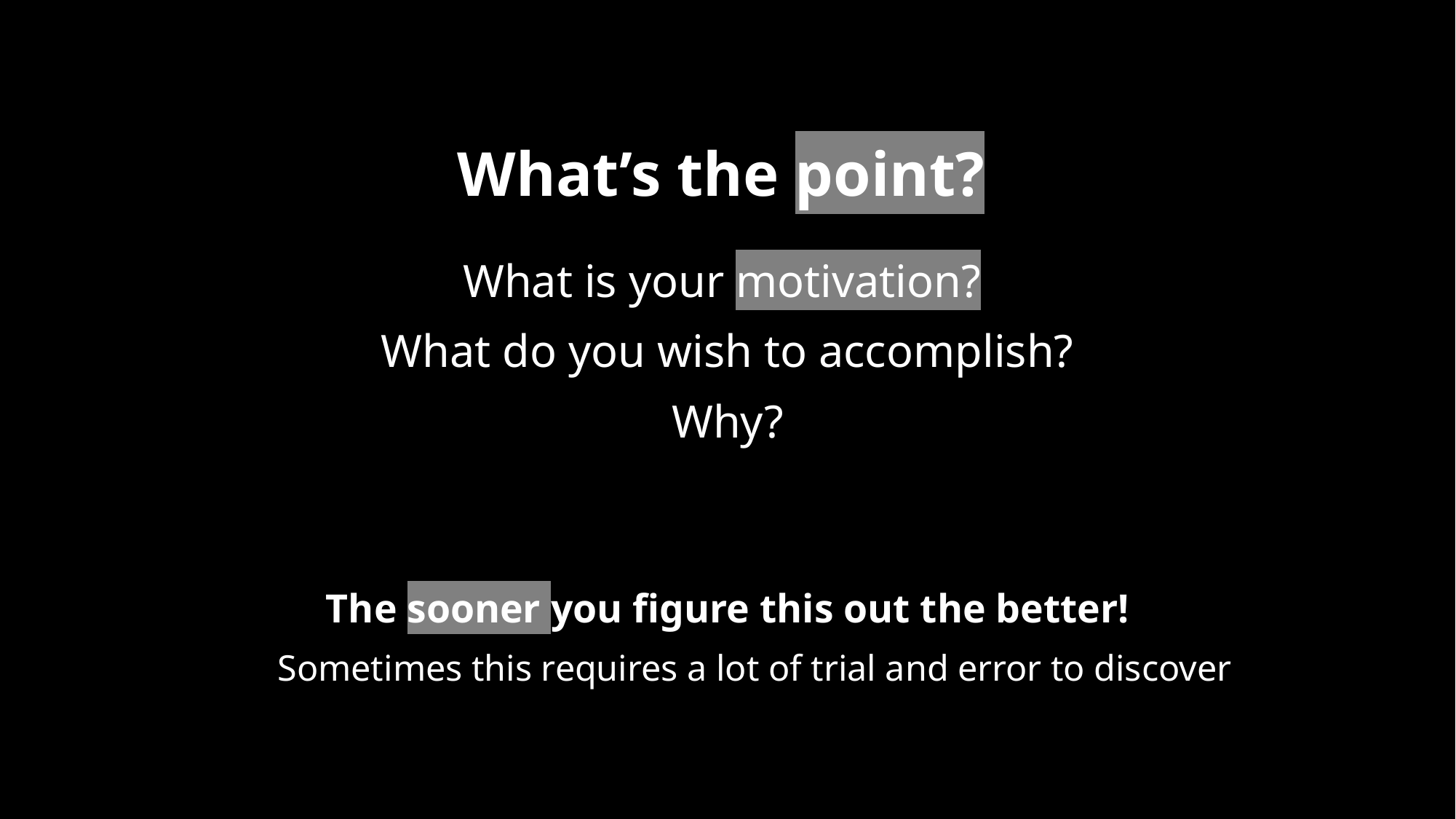

# What’s the point?
What is your motivation?
What do you wish to accomplish?
Why?
The sooner you figure this out the better!
Sometimes this requires a lot of trial and error to discover

## Slide 24
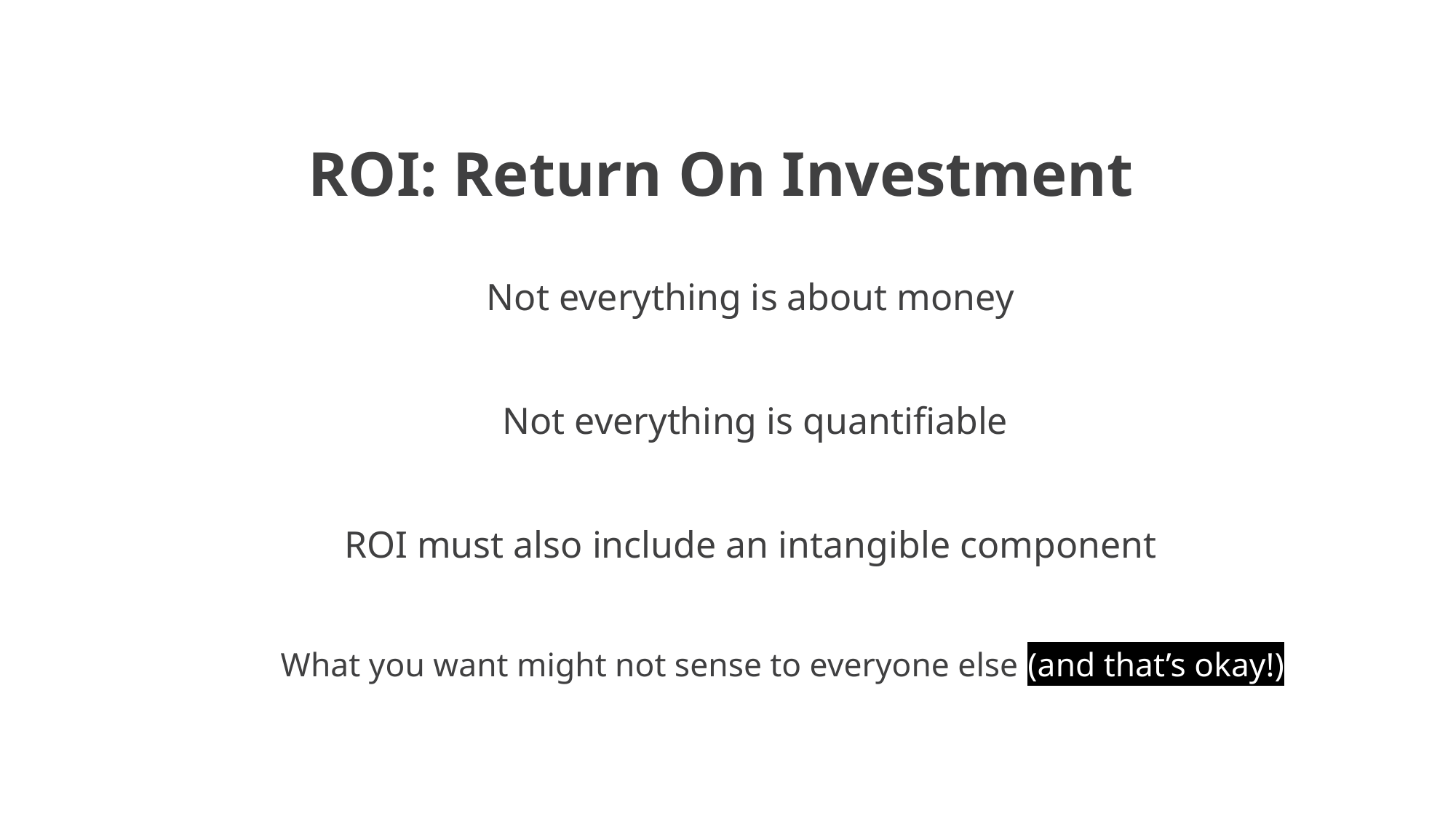

# ROI: Return On Investment
Not everything is about money
Not everything is quantifiable
ROI must also include an intangible component
What you want might not sense to everyone else (and that’s okay!)

## Slide 25
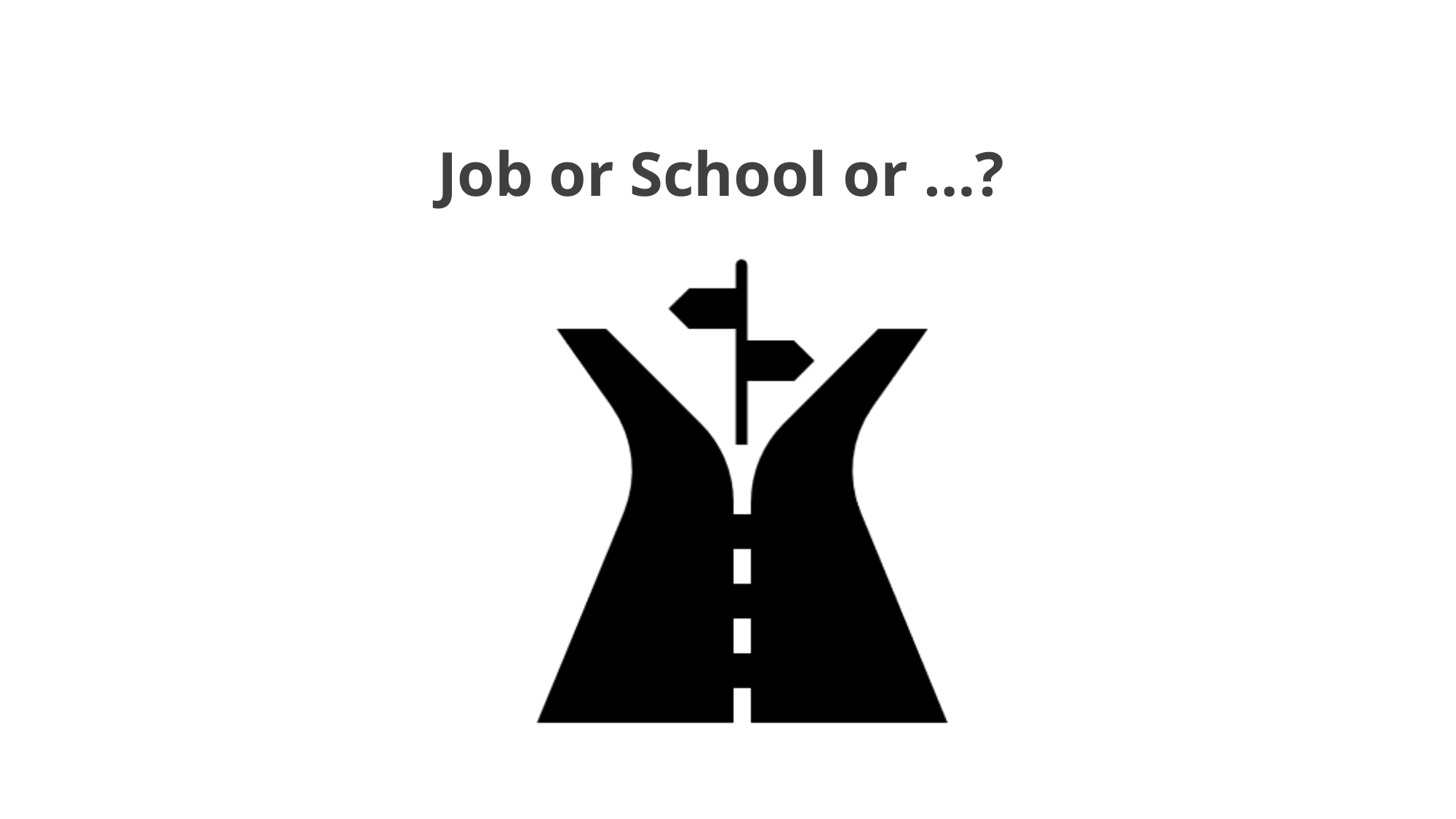

# Job or School or …?
This Photo by Unknown Author is licensed under CC BY-NC-ND
This Photo by Unknown Author is licensed under CC BY-SA

## Slide 26
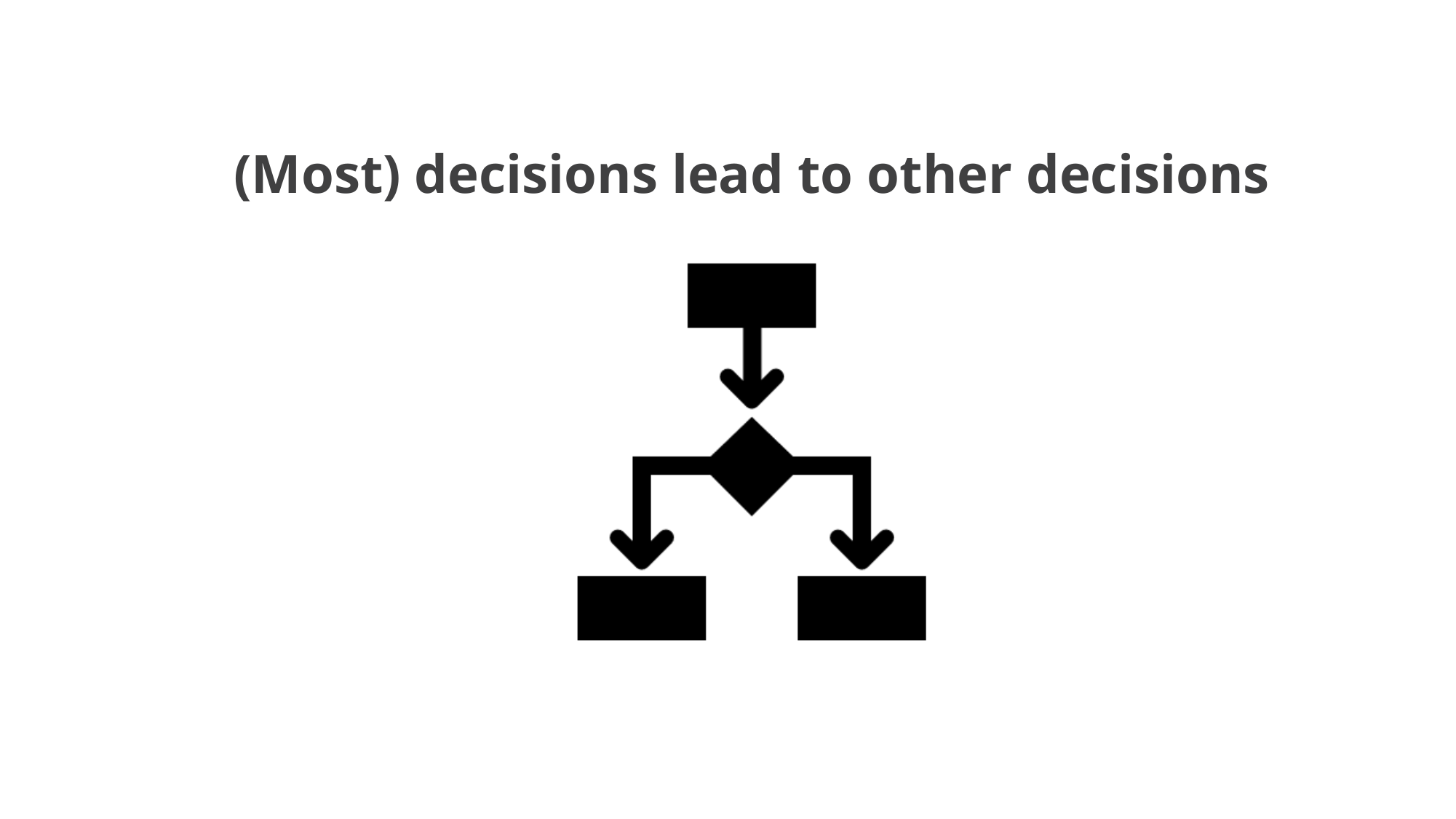

# (Most) decisions lead to other decisions

## Slide 27
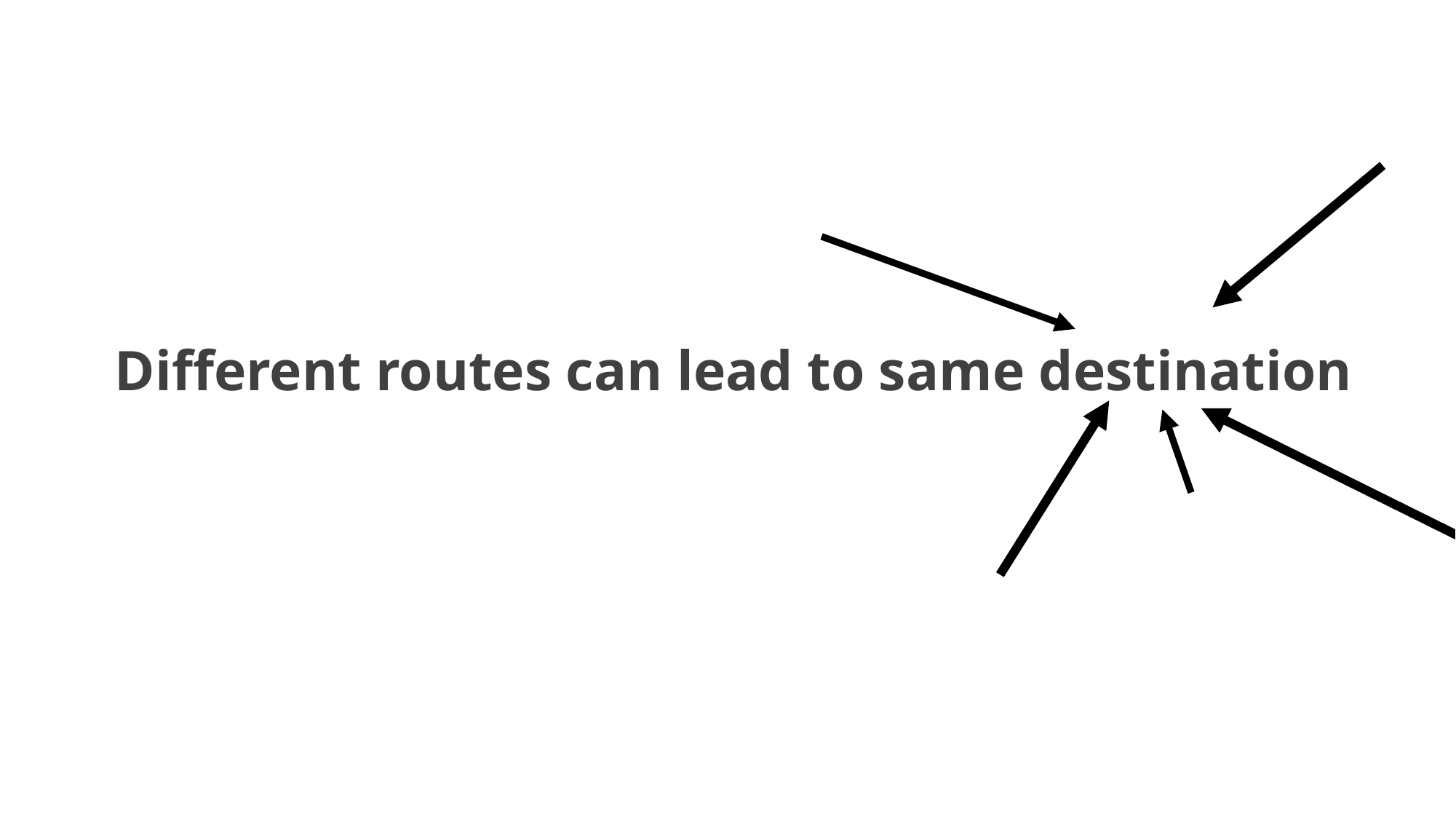

# Different routes can lead to same destination

## Slide 28
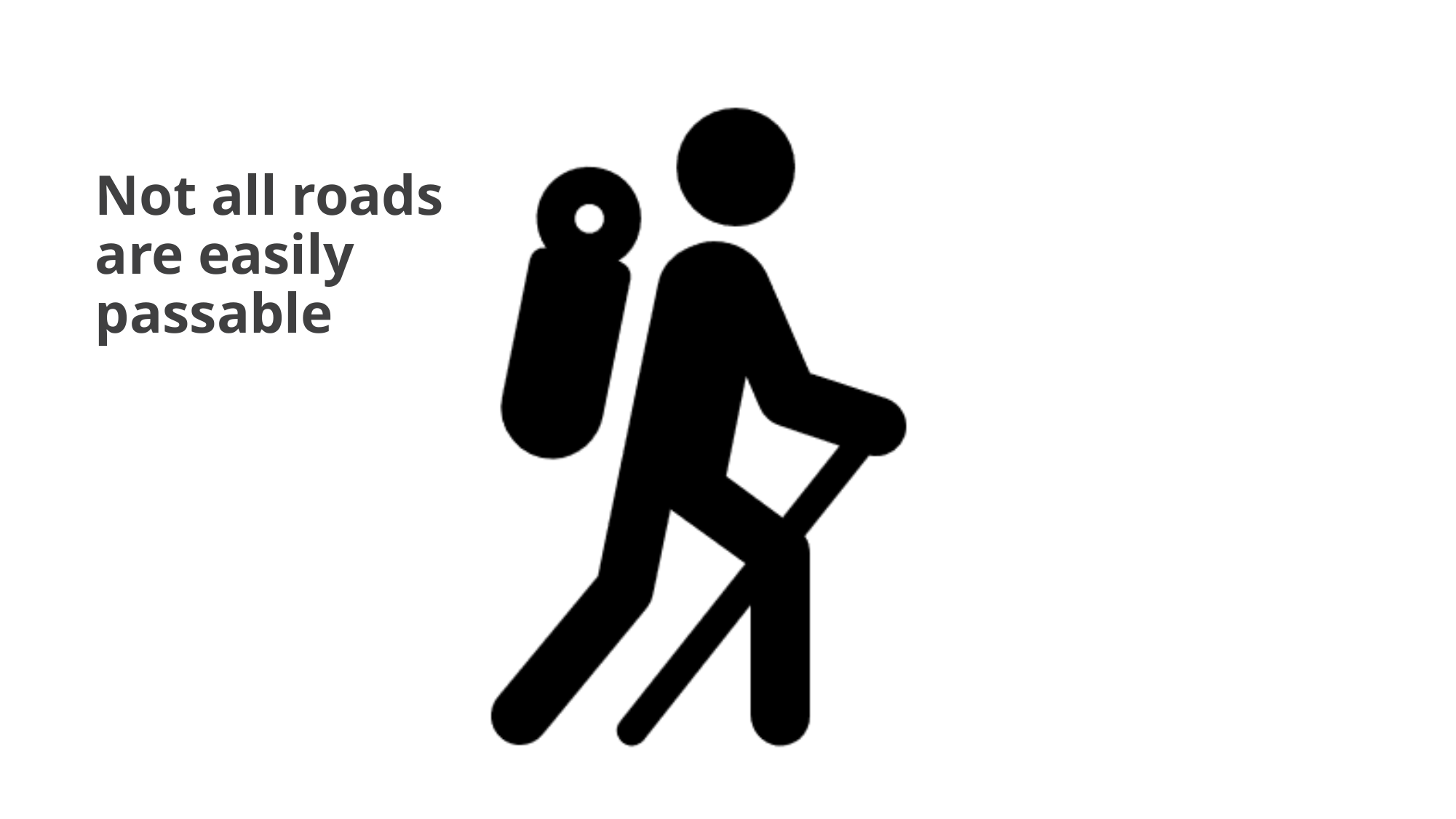

# Not all roads are easily passable

## Slide 29
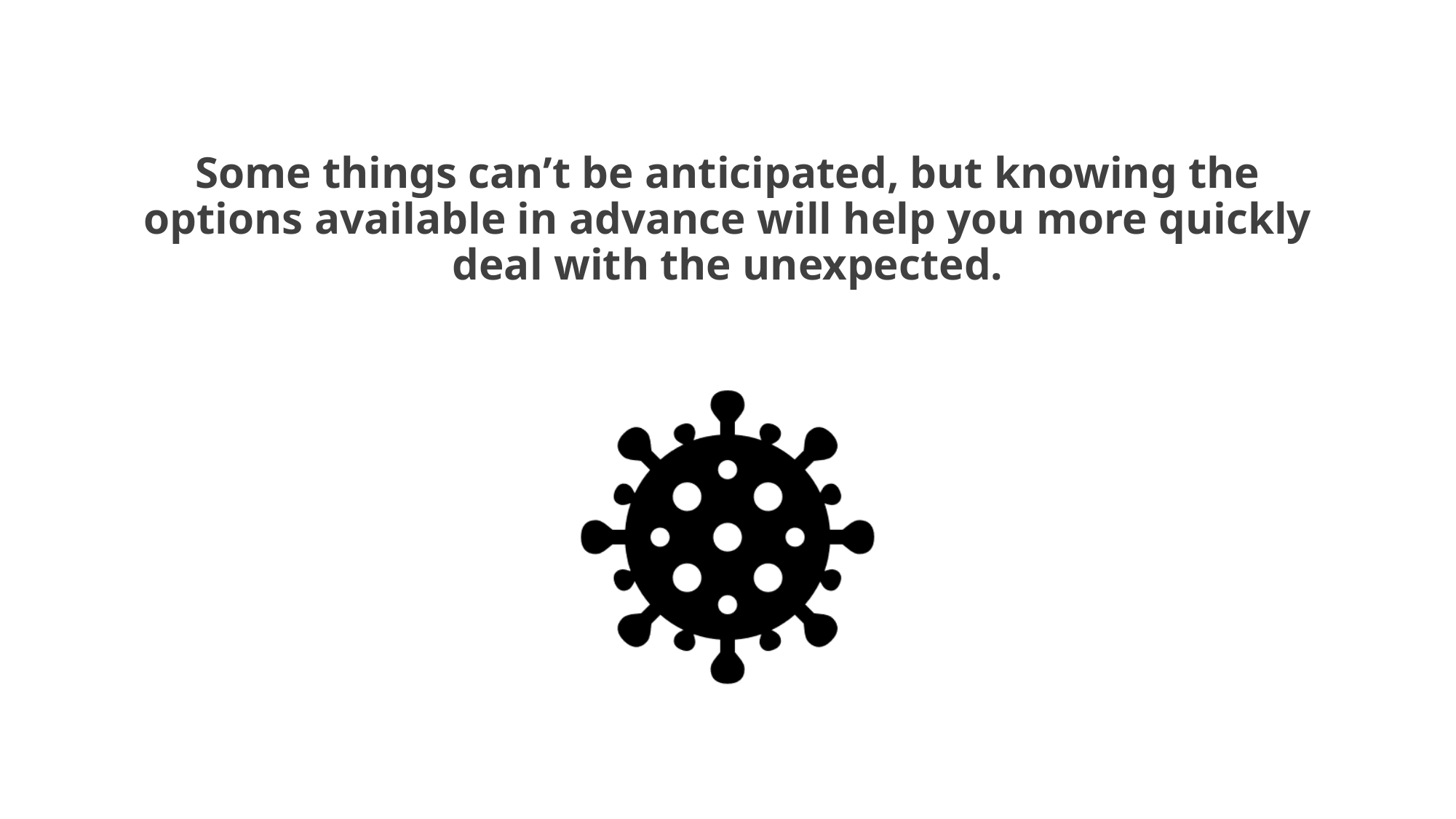

# Some things can’t be anticipated, but knowing the options available in advance will help you more quickly deal with the unexpected.

## Slide 30
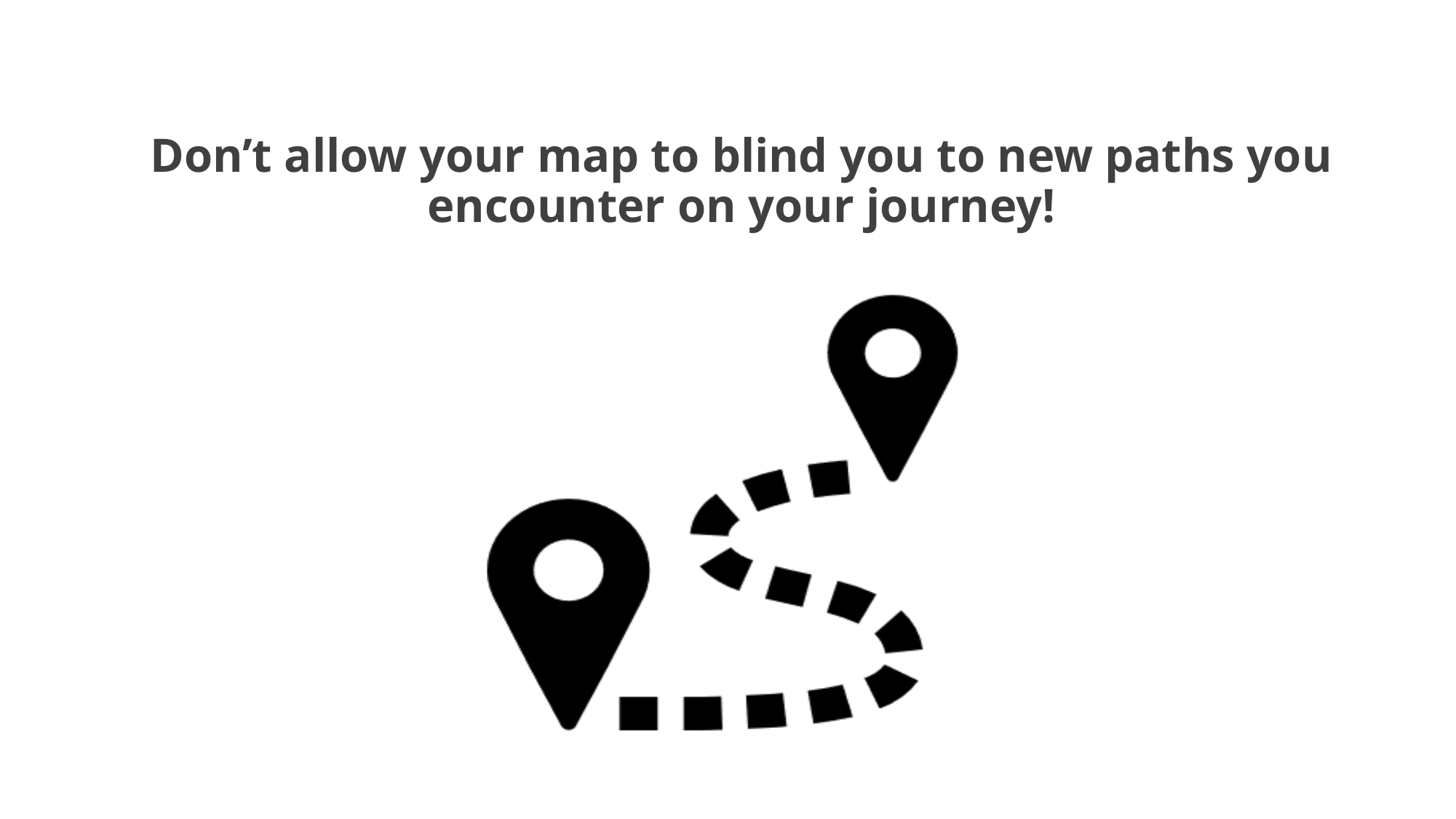

# Don’t allow your map to blind you to new paths you encounter on your journey!

## Slide 31
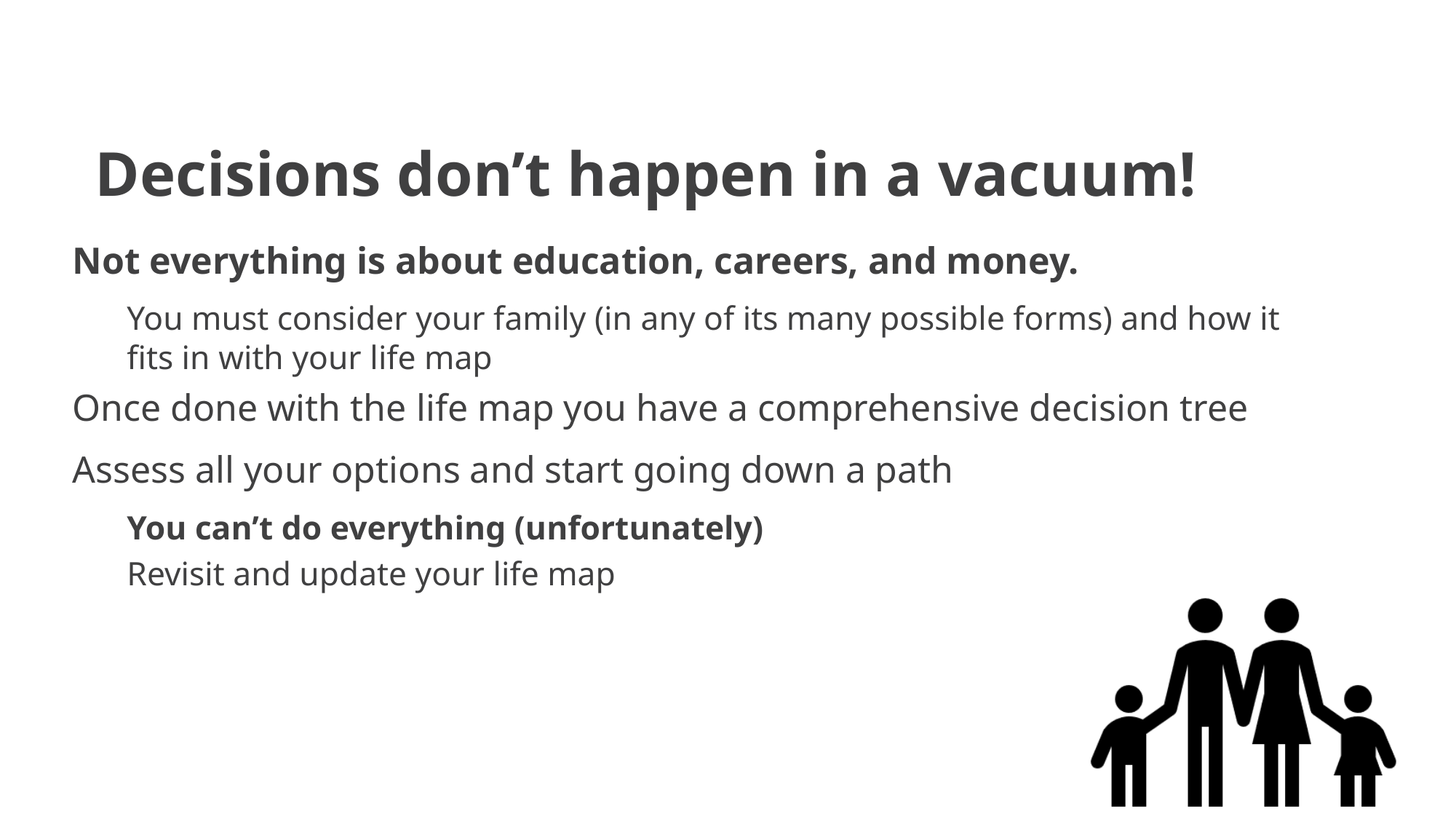

# Decisions don’t happen in a vacuum!
Not everything is about education, careers, and money.
You must consider your family (in any of its many possible forms) and how it fits in with your life map
Once done with the life map you have a comprehensive decision tree
Assess all your options and start going down a path
You can’t do everything (unfortunately)
Revisit and update your life map

## Slide 32
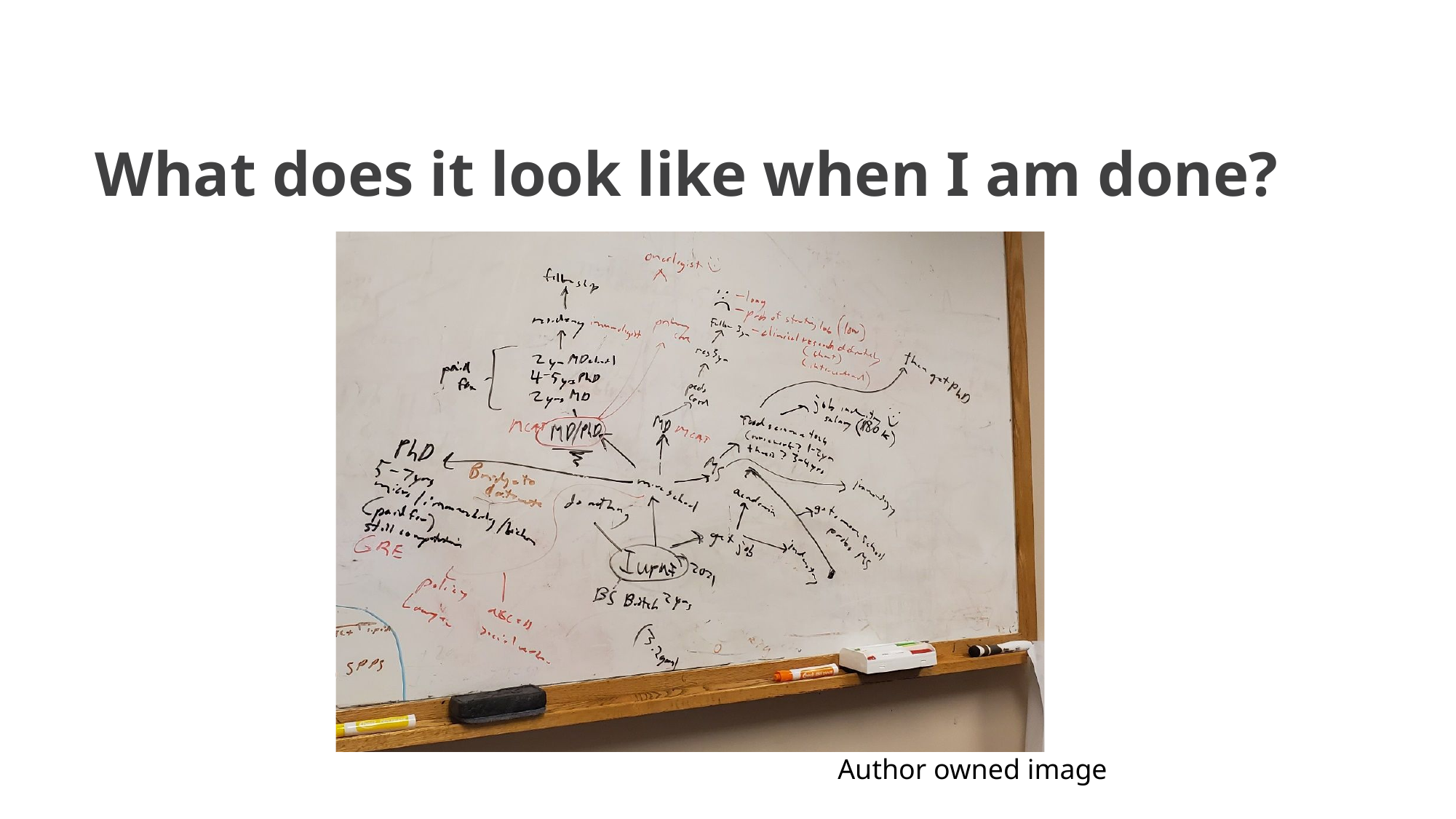

# What does it look like when I am done?
Author owned image

## Slide 33
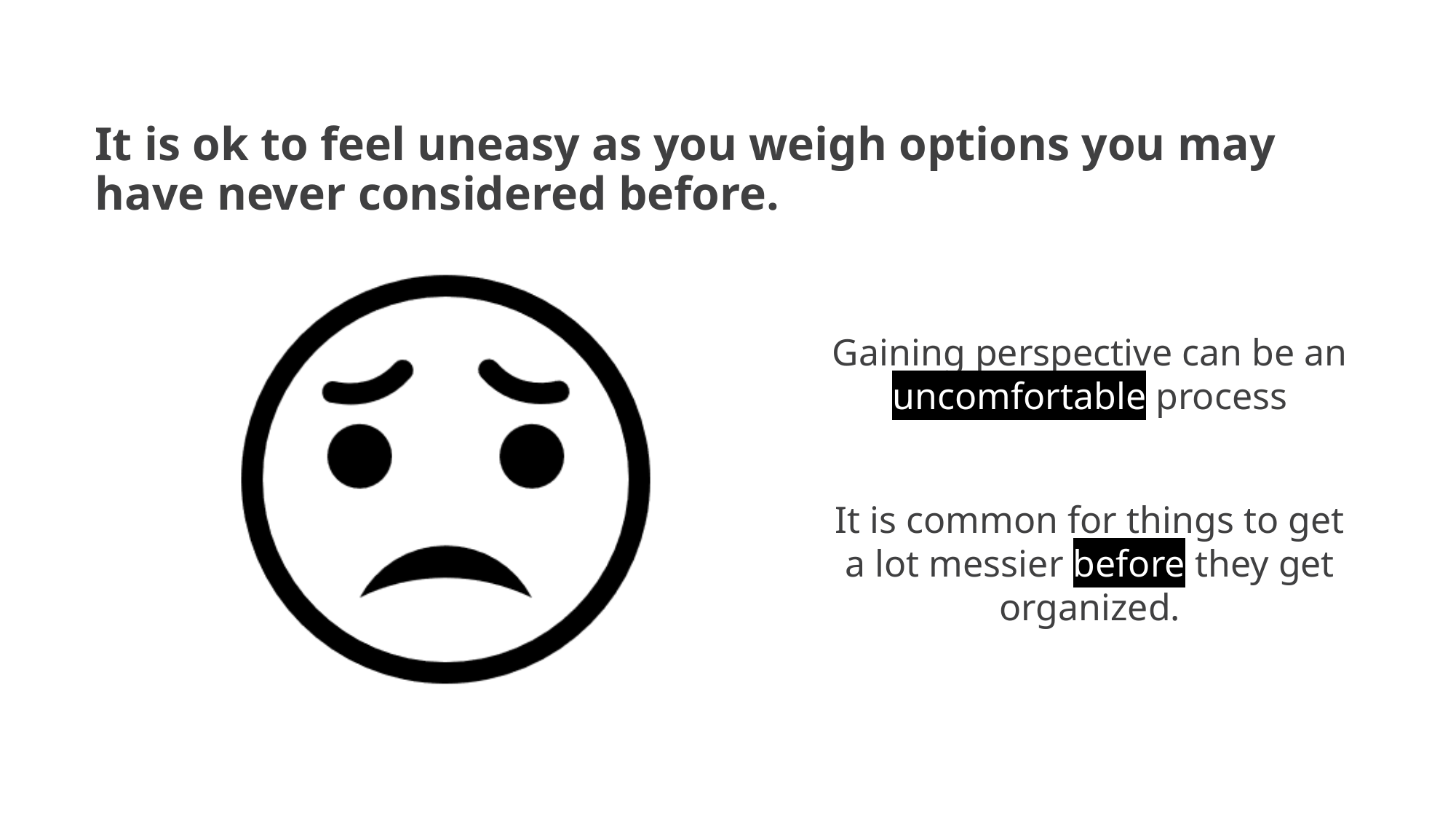

# It is ok to feel uneasy as you weigh options you may have never considered before.
Gaining perspective can be an uncomfortable process
It is common for things to get a lot messier before they get organized.

## Slide 34
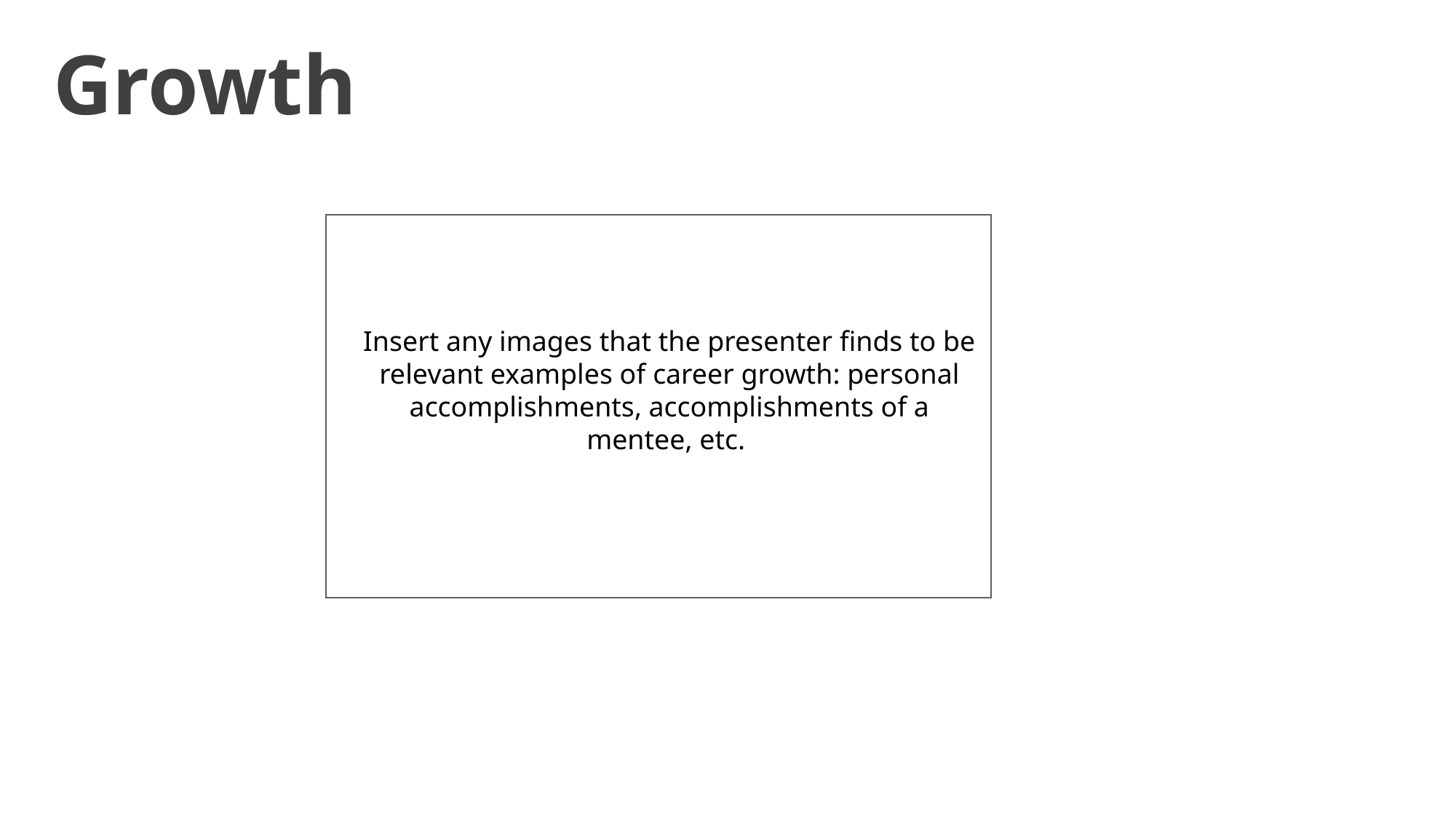

# Growth
Insert any images that the presenter finds to be relevant examples of career growth: personal accomplishments, accomplishments of a mentee, etc.

## Slide 35
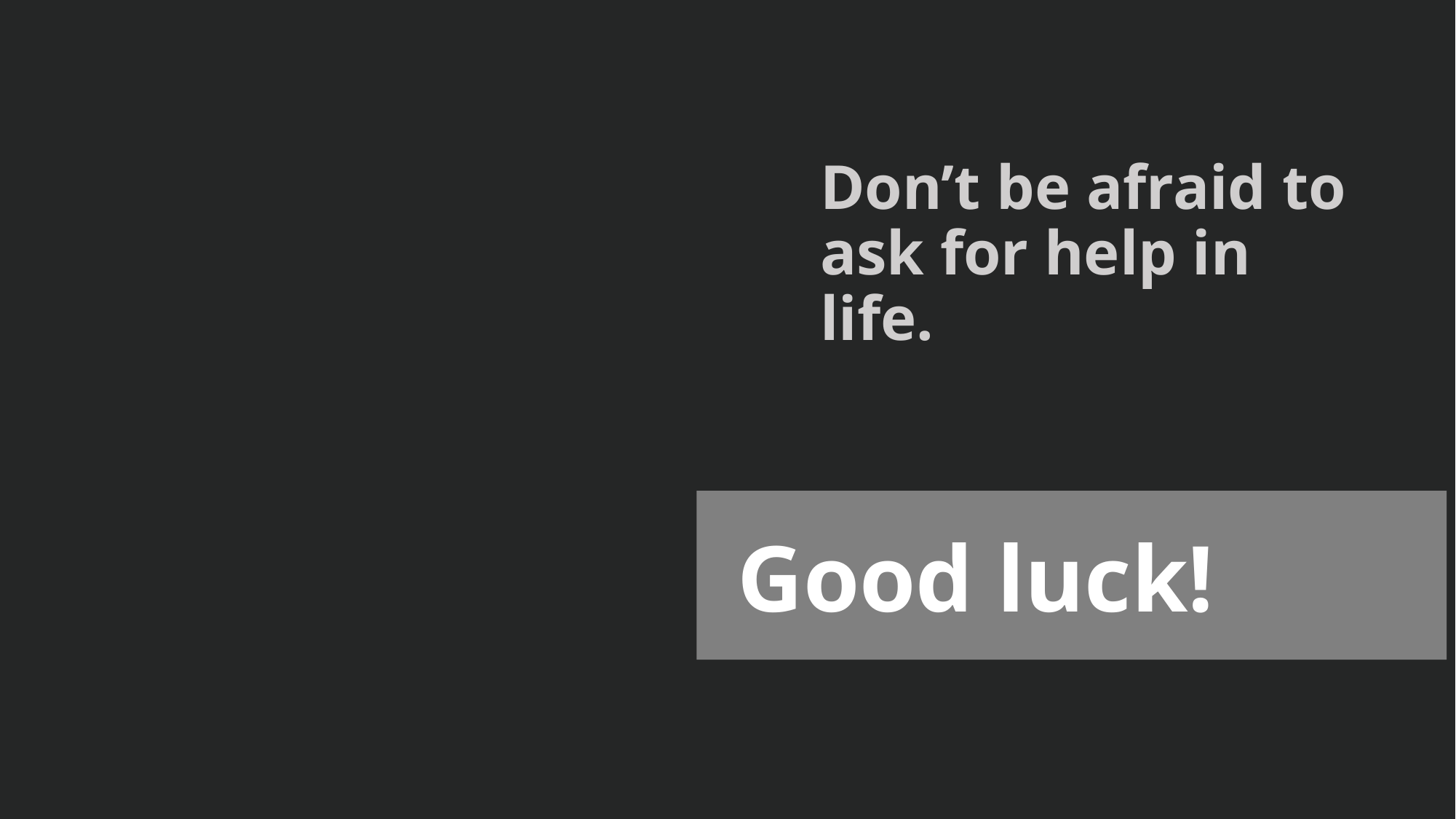

Don’t be afraid to ask for help in life.
Good luck!
