# Supplementary material for: Use of the “Future Life Map” exercise to improve awareness of career options and opportunities in underrepresented minority undergraduate students pursuing STEM careers
Source: PLoS One. 2022 Feb 10;17(2):e0263848. doi: 10.1371/journal.pone.0263848 (PMC8830657; doi:10.1371/journal.pone.0263848)
Supplement: S3 Appendix — (DOCX) [file pone.0263848.s003.docx]

**Appendix C.**

Discussion Questions for Session #2

1. What are examples of opportunities you included?

*This might include different job, schools, degrees*

- 1. What other education did you consider?

*For example, consideration of PA or NP school for those who have previously considered MD/DO, considering masters instead of PhD*

- 1. Which jobs/careers did you consider?

*May include consideration of jobs on the path to the dream job, not just the dream job identified*

- - 1. Was academia vs industry a consideration?

*Participants may report not having thought much about this differentiation prior to the exercise*

- 1. Did anyone consider dropping out/switching to an entirely new path?

*Some participants might identify negative consequences to dropping out of their current path (eg financial implications), others may report considering pursuing unrelated interests/hobbies*

- 1. Did you consider what options you have if your current path is not successful? Did you prepare for the possibility of failure?

*Ask participants if their future life maps include a detailed “back-up plan” or “alternative routes”*

1. Were there things you considered that you had never considered previously?

*This might include different career paths not considered, or a potentially helpful/important decision-making factor not previously considered*

1. Did you/how did you take family into consideration when completing the map?

*Participants might have considered the importance of location, time with children, or significant other’s career, or family needs*

1. Did you/how did you consider “return on investment (ROI)” when completing the map?

*Money, time, altruism might be common discussion points*

1. What did you think of the exercise?
   1. What did you find helpful?
   2. Do you feel empowered by the exercise?
   3. Would you recommend this to other people?
