# Supplementary material for: Use of the “Future Life Map” exercise to improve awareness of career options and opportunities in underrepresented minority undergraduate students pursuing STEM careers
Source: PLoS One. 2022 Feb 10;17(2):e0263848. doi: 10.1371/journal.pone.0263848 (PMC8830657; doi:10.1371/journal.pone.0263848)
Supplement: S4 Appendix — (DOCX) [file pone.0263848.s004.docx]

**Appendix D.**

Future Life Map Post Survey

Thank you for completing The Life Map module. Please complete this brief survey to help improve this module for future use.

Gender?

- Female
- Male
- Other
- Prefer not to say

Age?

- FREE TEXT

Academic year/major?

- FREE TEXT

Have you ever performed an exercise similar to this previously?

- Yes
- No

Please describe the exercise similar to The Life Map that you've completed.

- FREE TEXT

Rate the quality of information provided in the Life Map pdf:

1 2 3 4 5

1= not very informative, 5= very informative

Rate the quality of information provided in the Life Map presentation:

1 2 3 4 5

1= not very informative, 5= very informative

How informed on future life decisions did you feel PRIOR to performing the Life Map exercise?

1 2 3 4 5

1= not very informed, 5= very informed

How informed on future life decisions do you feel AFTER performing the Life Map exercise?

1 2 3 4 5

1= not very informed, 5= very informed

Did this exercise help you to consider alternative options that you did not consider previously?

- Yes
- No

Did this exercise help you to reconsider an option that you previously ruled out?

- Yes
- No

Did this exercise help you to learn new details about what your motivation is?

- Yes
- No

Do you feel more empowered regarding what you will do next in life?

1 2 3 4 5

1= not empowered, 5= very empowered

What was the most valuable part of this exercise?

- FREE TEXT

Which aspects of the course worked well?

- FREE TEXT

Would you recommend this module to others?

1 2 3 4 5

1= would not recommend, 5= highly likely to recommend

How likely are you to repeat the Life Map exercise in the future to help in your decision-making process?

1 2 3 4 5

1= not at all likely, 5= highly likely

How could the Life Map module be improved?

- FREE TEXT

Any other comments?

- FREE TEXT
