# Supplementary material for: Use of the “Future Life Map” exercise to improve awareness of career options and opportunities in underrepresented minority undergraduate students pursuing STEM careers
Source: PLoS One. 2022 Feb 10;17(2):e0263848. doi: 10.1371/journal.pone.0263848 (PMC8830657; doi:10.1371/journal.pone.0263848)
Supplement: S5 Appendix — (DOCX) [file pone.0263848.s005.docx]

**Supplementary Data:**

**Use of the “Future Life Map” exercise to improve awareness of career options and opportunities in underrepresented minority undergraduate students pursuing STEM careers**

**Anne Whitehead MD^a^, Nathan J. Alves PhD^a*^**

**^a^ Department of Emergency Medicine, Indiana University School of Medicine, Indianapolis, IN**

***Corresponding author**

**Nathan J Alves, PhD**

**Indiana University School of Medicine**

**635 Barnhill Dr. Rm. 2063**

**Indianapolis, IN 46202 United States of America**

**nalves@iu.edu**

**Table 1:** Raw Survey Data. All questions that were not yes/no were on a 5-point Likert Scale.

| **Subject** | **Gender** | **Age** | **1** | **2** | **3** | **4** | **5** | **6** | **7** | **8** | **9** | **10** | **11** |
| --- | --- | --- | --- | --- | --- | --- | --- | --- | --- | --- | --- | --- | --- |
| **1** | Male | 19 | No | 5 | 4 | 4 | 5 | Yes | No | No | 3 | 4 | 4 |
| **2** | Male | 20 | No | 5 | 5 | 5 | 5 | Yes | Yes | Yes | 5 | 5 | 5 |
| **3** | Female | 19 | No | 4 | 4 | 3 | 4 | Yes | No | Yes | 3 | 3 | 4 |
| **4** | Female | 17 | No | 5 | 5 | 3 | 4 | Yes | Yes | No | 3 | 4 | 4 |
| **5** | Male | 19 | No | 3 | 5 | 2 | 4 | Yes | Yes | Yes | 4 | 5 | 3 |
| **6** | Male | 19 | No | 5 | 5 | 5 | 5 | Yes | Yes | Yes | 5 | 5 | 5 |
| **7** | Female | 19 | No | 2 | 3 | 4 | 4 | No | No | Yes | 3 | 2 | 2 |
| **8** | Female | 19 | Yes | 5 | 4 | 3 | 4 | Yes | Yes | Yes | 5 | 5 | 4 |
| **9** | Female | 18 | No | 4 | 4 | 2 | 3 | No | Yes | Yes | 3 | 4 | 5 |
| **10** | Female | 19 | No | 4 | 5 | 2 | 4 | Yes | Yes | yes | 3 | 4 | 3 |
| **11** | Female | 19 | No | 5 | 4 | 2 | 4 | Yes | Yes | Yes | 4 | 5 | 5 |
| **12** | Male | 21 | No | 5 | 5 | 2 | 4 | Yes | Yes | Yes | 3 | 4 | 5 |
| **13** | Female | 19 | No | 5 | 5 | 4 | 4 | Yes | Yes | No | 5 | 5 | 5 |
| **14** | Female | 20 | Yes | 4 | 5 | 3 | 4 | yes | No | Yes | 4 | 5 | 5 |
| **15** |  |  | No | 3 | 5 | 4 | 2 | Yes | Yes | Yes | 4 | 5 | 4 |
| **16** |  |  | No | 4 | 4 | 2 | 4 | Yes | Yes | Yes | 4 | 4 | 4 |
| **17** |  |  | No | 5 | 4 | 5 | 4 | Yes | Yes | Yes | 4 | 5 | 4 |
| **18** |  |  | No | 5 | 5 | 3 | 5 | Yes | Yes | Yes | 3 | 4 | 4 |
| **19** | Male | 32 | Yes | 3 | 3 | 4 | 4 | No | No | No | 3 | 4 | 3 |
| **20** | Female | 22 | Yes | 5 | 5 | 2 | 4 | Yes | Yes | Yes | 5 | 5 | 5 |
| **21** | Female | 20 | No | 3 | 4 | 2 | 4 | Yes | No | Yes | 3 | 4 | 4 |
| **22** | Female | 22 | No | 5 | 5 | 2 | 4 | Yes | Yes | Yes | 4 | 5 | 3 |
| **23** | Female | 23 | No | 5 | 5 | 2 | 5 | Yes | Yes | Yes | 5 | 5 | 5 |
| **24** | Female | 23 | No | 5 | 5 | 2 | 4 | Yes | Yes | Yes | 4 | 5 | 5 |
| **25** | Male | 23 | No | 5 | 5 | 3 | 4 | Yes | Yes | Yes | 4 | 5 | 4 |
| **26** | Male | 23 | No | 4 | 4 | 3 | 3 | Yes | No | Yes | 2 | 4 | 4 |
| **27** | Female | 23 | No | 4 | 5 | 2 | 4 | Yes | Yes | Yes | 4 | 5 | 5 |

Corresponding question numbers:

1. Have you ever performed an exercise similar to this previously?
2. Rate the quality of information provided in the Life Map pdf?
3. Rate the quality of information provided in the Life Map presentation?
4. How informed on future life decisions did you feel PRIOR to performing the Life Map exercise?
5. How informed on future life decisions do you feel AFTER performing the Life Map exercise?
6. Did this exercise help you to consider alternative options that you did not consider previously?
7. Did this exercise help you to reconsider an option that you previously ruled out?
8. Did this exercise help you to learn new details about what your motivation is?
9. Do you feel more empowered regarding what you will do next in life?
10. Would you recommend this module to others?
11. How likely are you to repeat the Life Map exercise in the future to help in your decision-making process?


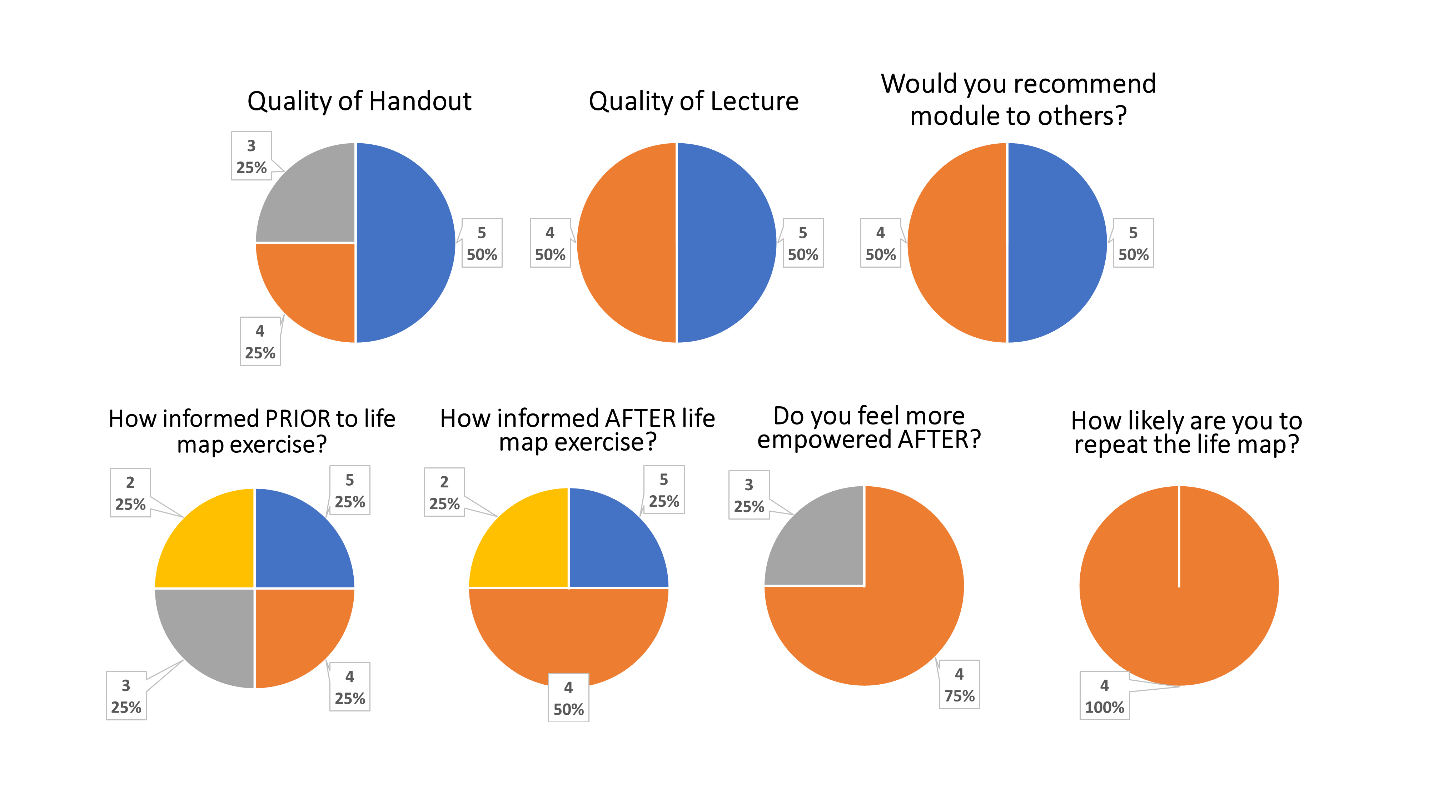


**Figure 1:** Pie charts containing the percent prevalence of survey responses corresponding to the group of 4 individuals that did not specify a gender. This data is included in the overall data pie charts present in the main text but was not specifically called out as a subpopulation due to the relatively small number in this group.

**Table 2:** Likert scores were grouped by 4 and 5 vs 1 and 2 and 3 to differentiate highly rated vs mid/low rated scores. The percent of total respondents is included and the data is presented as overall data and split by gender.

|  | **Likert Score Groups** | **Quality of Handout** | **Quality of Lecture** | **How informed PRIOR to life map exercise?** | **How informed AFTER life map exercise?** | **Do you feel more empowered AFTER?** | **Would you recommend module to others?** | **How likely are you to repeat the life map?** |
| --- | --- | --- | --- | --- | --- | --- | --- | --- |
| **Overall** | **percent 4+5** | 81 | 93 | 30 | 89 | 59 | 93 | 81 |
| (n=27) | **percent 1+2+3** | 19 | 7 | 70 | 11 | 41 | 7 | 19 |
|  |  |  |  |  |  |  |  |  |
| **Female** | **percent 4+5** | 87 | 93 | 13 | 93 | 60 | 87 | 80 |
| (n=15) | **percent 1+2+3** | 13 | 7 | 87 | 7 | 40 | 13 | 20 |
|  |  |  |  |  |  |  |  |  |
| **Male** | **percent 4+5** | 75 | 88 | 50 | 88 | 50 | 100 | 75 |
| (n=8) | **percent 1+2+3** | 25 | 13 | 50 | 13 | 50 | 0 | 25 |
|  |  |  |  |  |  |  |  |  |
| **Other** | **percent 4+5** | 75 | 100 | 50 | 75 | 75 | 100 | 100 |
| (n=4) | **percent 1+2+3** | 25 | 0 | 50 | 25 | 25 | 0 | 0 |
